# Identification of biomarkers and mechanism exploration of ferroptosis related genes regulated by m6A in type 2 diabetes mellitus

**DOI:** 10.1186/s41065-025-00385-9

**Published:** 2025-02-18

**Authors:** Jing Wang, Xuying Li, Juan Geng, Ruiduo Wang, Gang Ma, Pan Liu

**Affiliations:** 1 Department of Anaesthesiology, Northwest Women’s and Children’s Hospital, Yanxiang Road, Yanta District, Xi’an, 710000 Shanxi Province China; 2 Department of Anesthesiology, Hainan General Hospital, Hainan Affiliated Hospital of Hainan Medical University, Haikou, Hainan China; 3 State Key Laboratory of Transient Optics and Photonics, Xi’an Institute of Optics and Precision Mechanics, Chinese Academy of Science, Xi’an, 710119 China; 4 Department of Anesthesiology, General Hospital of Ningxia Medical University, Yinchuan, China 704 Shengli Street, Yinchuan, 750004 Ningxia China; 5 Department of Neurology, Renmin Hospital, Hubei University of Medicine, Maojian District, No. 39, Chaoyang Middle Road, Shiyan, Hubei 442000 People’s Republic of China

**Keywords:** Differentially expressed genes, Diagnostic model, Gene Set Enrichment Analysis (GSEA), Biomarkers, Molecular docking

## Abstract

**Purpose:**

This study is aims to explore the role of ferroptosis genes regulated by N6-methyladenosine (m6A) in Type 2 diabetes mellitus (T2DM).

**Material and methods:**

Firstly, differentially expressed m6A-FRGs (DEm6A-FRGs) were obtained by intersecting the differentially expressed genes (DEGs) and the m6A-related ferroptosis genes (m6A-FRGs). After enrichment analysis of DEm6A-FRGs, artificial neural network (ANN) and nomogram models were constructed using 4 biomarkers. Moreover, the gene set enrichment analysis of biomarkers was performed. Furthermore, the transcription factors (TF)-mRNA and competing endogenous RNAs (ceRNA) regulatory networks were constructed to reveal the potential regulation of biomarkers at molecular level. In addition, the targeted drugs of biomarkers were predicted, and the molecular docking was used to study the inter-molecular interactions between biomarkers and targeted drugs by “AutoDockvina”.

**Results:**

Totals of 10 DEm6A-FRGs were obtained by intersecting the 402 DEGs and 299 m6A-FRGs. Moreover, the ANN model and nomogram model were constructed with 4 biomarkers including CDKN1A, MIOX, MYCN and CD82, among them, CDKN1A was the most important biomarker for forecasting T2DM. Notably, the function of extracellular matrix structural constituent was low expression in CD82 and MIOX, the function of mitochondrial protein-containing complex was high expression in CD82 and CDKN1A. Furthermore, TP63 could regulate CD82, CDKN1A and MIOX, GATA3 could regulate CD82, CDKN1A and MYCN at the same time. The ceRNA network was constructed with 4 mRNAs, 51 miRNAs and 37 lncRNAs, among them, XIST was a key lncRNA that associated with 12 miRNAs, which could influence CDKN1A. In addition, bisphenol A was associated with CD82 and MYCN, CGP 25608 was associated with CDKN1A and MIOX.

**Conclusion:**

This study revealed the potential molecular mechanisms of m6A-related ferroptosis genes in T2DM, which could provide novel insights for the clinical diagnosis and treatment of T2DM.

**Supplementary Information:**

The online version contains supplementary material available at 10.1186/s41065-025-00385-9.

## Introduction

Type 2 diabetes mellitus (T2DM) is a prevalent metabolic disease that is commonly associated with obesity, high blood pressure, high cholesterol, and other metabolic diseases such as cardiovascular disease [1, 2]. It is primarily characterized by insulin resistance and islet beta cells dysfunction [3]. Treatments for T2DM include lifestyle changes (such as modifications in diet and exercise habits), medications (such as oral medications and insulin injections), and surgical treatments (such as gastrointestinal bypass surgery) [3]. Searching for biomarkers could assist doctors in identifying early signs of diabetes, intervening and treating those at high risk early, thus reducing the incidence of complications. Furthermore, searching for biomarkers can also be used to evaluate the effectiveness and prognosis of treatment for T2DM, and provide reference for personalized treatment.

N6-methyladenosine (m6A) modification is a highly prevalent RNA modification that has been shown to play a critical role in numerous biological processes, including post-transcriptional regulation, RNA splicing, RNA stability, and RNA translation [4–6].Changes in m6A modification levels have been shown to regulate insulin signaling pathways, glucose metabolism, insulin secretion, and the function of islet beta cells, thereby influencing insulin resistance and the development of T2DM [7–9]. Ferroptosis is a newly recognized type of programmed cell death that has gained increasing attention in recent years [10]. Research has demonstrated that ferroptosis plays a crucial role in the pathogenesis of T2DM, and serum iron levels are positively correlated with blood glucose levels in T2DM patients [11, 12]. However, the role of ferroptosis genes regulated by m6A in T2DM has not yet been fully explored.

In this study, we integrated literature-reported m6A regulatory genes, ferroptosis related genes from the FerrDb database, and T2DM transcriptome data from the Gene Expression Omnibus (GEO) database to identify m6A ferroptosis genes. Then we used machine learning algorithms to identify four biomarkers of T2DM. Additionally, we constructed transcription factors (TF)-mRNA regulatory and competing endogenous RNAs (ceRNA) networks of these biomarkers to predict potential drugs, which can provide potential targets for clinical diagnosis of T2DM and a theoretical basis for further understanding of the driving mechanism of T2DM.

## Material and methods

### Data extraction

T2DM-related datasets GSE76895 (GPL570) and GSE41762 (GPL6244) were extracted from GEO database (https://www.ncbi.nlm.nih.gov/geo/). Among them, GSE76895 was used as the training dataset, which includes pancreatic islet samples of 36 T2DM and 32 healthy controls (HC), GSE41762 was used as the validation dataset to verify the expression levels of biomarkers, which includes pancreatic islet samples of 20 T2DM and 57 HC. 621 ferroptosis-related genes were selected from the FerrDb database (http://www.zhounan.org/ferrdb/current), and 23 m6A methylation regulatory-related genes were downloaded from previous literature [13].

### Function analysis of differentially expressed DEm6A-FRGs of T2DM

Firstly, the mRNA expression levels between 36 T2DM and 32 HC samples were compared by the “limma” R package (version 3.52.2) (|log_2_FC|> 0.5, *p* < 0.05) [14]. The correlation of 23 m6A methylation regulatory-related genes and 621 ferroptosis-related genes were calculated by “Spearman”, and the m6A-related ferroptosis genes (m6A-FRGs) were obtained with |Cor|> 0.3 and *p* < 0.05. Then, the differentially expressed m6A-FRGs (DEm6A-FRGs) were obtained by intersecting the differentially expressed genes (DEGs) and m6A-FRGs. Besides, the function analyses of DEm6A-FRGs were conducted by “clusterprofiler” R package (version 4.4.4) [15].

### Construction of the diagnostic models

In this study, random forest (RF) and support vector machine recursive feature elimination (SVM-RFE) methods were used to obtain the importance ranking of DEm6A-FRGs and the error rate and accuracy of each iteration combination, respectively. The best combination with lowest error rate was selected, and the corresponding genes were taken as the characteristic/feature genes, respectively [16]. Then, the biomarkers were screened by intersecting the characteristic genes and feature genes for subsequent analysis.

Artificial neural network (ANN) model and nomogram model were constructed for clinical diagnosis of T2DM. On the one hand, the data was standardized using the “neuralnet” R package (version 1.44.2) [17] with the parameters preProcess = c("scale", "center"), and the output was calculated as a probability using the logistic activation function (act.fct = "logistic"). The number of hidden neurons was set to 3, constructing the ANN model. The weight of biomarkers was determined in the training set (GSE76895), and the classification efficiency of the model constructed using the expression and weight of biomarkers was evaluated. Additionally, the performance and stability of the ANN model were evaluated using fivefold cross-validation with number = 5 and repeats = 8, effectively reducing the risk of overfitting caused by insufficient data samples or random splitting. Meanwhile, the model's generalization ability was further validated in the external validation set (GSE41762). Besides, the receiver operating characteristic curves (ROC) were drew to assess the diagnose ability of ANN model. On the other hand, the logistic regression model (LRM) was constructed, and the nomogram model was constructed with these biomarkers by “RMS” R package (version 5.4–1) [18]. Then, the calibration curve, decision curve (DCA) and clinical impact curve were drawn to verify the validity of the nomogram.

### The gene set enrichment analysis (GSEA) of biomarkers

The correlation coefficient between each biomarker and all genes in GSE76895 were calculated, and Gene Set Enrichment Analysis (GSEA) of each biomarker was performed by “clusterProfiler” R package [19].

### Construction of TF-mRNA and ceRNA regulatory networks

Firstly, the potential TFs were screened in ChEA3 database (https://amp.pharm.mssm.edu/chea3/) and KnockTF database (http://www.licpathway.net/KnockTF/). Then, the targeted TFs were obtained by intersecting these 2 groups of potential TFs. Finally, the TF-mRNA network was constructed by “Cytoscape” (version 3.8.2) [20].

Furthermore, the potential miRNA were predicted in miRwalk database (http://mirwalk.umm.uni-heidelberg.de/) and mirtarBase database (https://mirtarbase.cuhk.edu.cn/). Then, the targeted miRNAs were obtained by intersecting these 2 groups of potential miRNAs. Next, the targeted lncRNAs were predicted in starbase database with clipExpNum > 3 (http://starbase.sysu.edu.cn/). Finally, the ceRNA network was constructed by “Cytoscape”.

### Drug prediction

The potential drugs were predicted by biomarkers in Comparative Toxicogenomics Database (CTD, http://ctdbase.org/), and the interaction relationships between potential drugs and biomarkers were visualized by “Cytoscape”. In addition, the targeted drugs which were associated with T2DM were further predicted in CDT. Besides, the interaction scores between biomarkers and T2DM were calculated.

Protein structure prediction of the biomarkers (MIOX, CD82, and MYCN) was performed using Alphafold3, and the structure with the highest prediction score was selected as the structure for subsequent molecular docking and molecular dynamics simulations (MDs). The protein structure of CDKN1A was obtained from the Research Collaboratory for Structural Bioinformatics Protein Data Bank (RCSB PDB, https://www.rcsb.org). The 3D structures of the targeted drugs were mind from the PubChem database (https://pubchem.ncbi.nlm.nih.gov), the protein structure of biomarkers were downloaded from the PDB. Water molecules and small molecule ligands were removed with “PyMOL” (v 2.5) [21] and the molecular docking was used to study the inter-molecular interactions between biomarkers and targeted drugs by “AutoDock Vina” (v 4.2) [22]. Specifically, the protein was hydrogenated and the charges were calculated using AutoDockTools (version 1.5.6) [23], and the charges of the small molecules were balanced, along with checking for rotatable bonds. Then, the docking box range was selected based on the receptor's active site. Finally, AutoDock Vina was used to calculate the receptor-ligand docking (num_modes = 20), and the structure with the lowest binding free energy from the output results was chosen (a docking binding energy < -5 kcal/mol indicates a good binding affinity).The “PyMol” was utilized to visualize results.

MDs is a molecular simulation method based on Newton's laws of motion for calculating time-dependent properties of molecular systems. To further validate the plausibility and reliability of the biomarker and drug docking results, in this study we performed 50 ns MDs through GROMACS2024.2 software. Protein sequences were retrieved from NCBI, screened in the database for human species, and AlphaFold3 predicted structures were selected as protein structure files. Parameter and topology files of protein and small molecule ligands were generated from AMBER14SB force field and AMNER gaff force field, respectively. The system was neutralized using NaCL at a concentration of 0.15 mol/L. The steepest descent method was used to minimize the energy consumption of the whole system. Prior to the MDs, the system temperature and pressure were stabilized. Specifically, simulations were performed using the NVT system at 300 K (temperature) and 100 ps to stabilize the temperature and the NPT system at 1 bar (pressure) and 100 ps to stabilize the pressure.

### Expression verification of biomarkers

The expression of biomarkers between the T2DM and HC samples were compared by “wilcox.test” in both GSE76895 and GSE41762 datasets.

### Western blotting

Primary pancreatic islet cells (CP-R015) were purchased from Procell Life Science&Technology Co.,Ltd. They were cultured with or without 30 mM glucose (high glucose, HG). Protein concentrations were extracted using ice-cold RIPA buffer (Beyotime, Nantong, China) containing protease inhibitor and phosphatase inhibitor (Thermo Fisher Scientific, Waltham, MA, USA). A 20–30 μg protein was subjected to 10%-15% SDS polyacrylamide gel electrophoresis and transferred onto polyvinylidene difluoride membranes (PVDF), Millipore, Bedford, MA, USA). The PVDF membranes were blocked in 5% skim milk for 1 h and then incubated overnight at 4℃ with CDKN1A (sc-6246, Santa Cruz Biotechnology), MYCN (sc-53993, Santa Cruz Biotechnology), MIOX (sc-376080, Santa Cruz Biotechnology) or CD82 (sc-518002, Santa Cruz Biotechnology). After incubating with appropriate secondary antibodies for 2 h, the band densities were determined using Image J software (NIH, Maryland, USA) and normalized to each internal control.

## Results

### Totals of 10 DEm6A-FRGs were obtained in T2DM

**Fig. 1:**
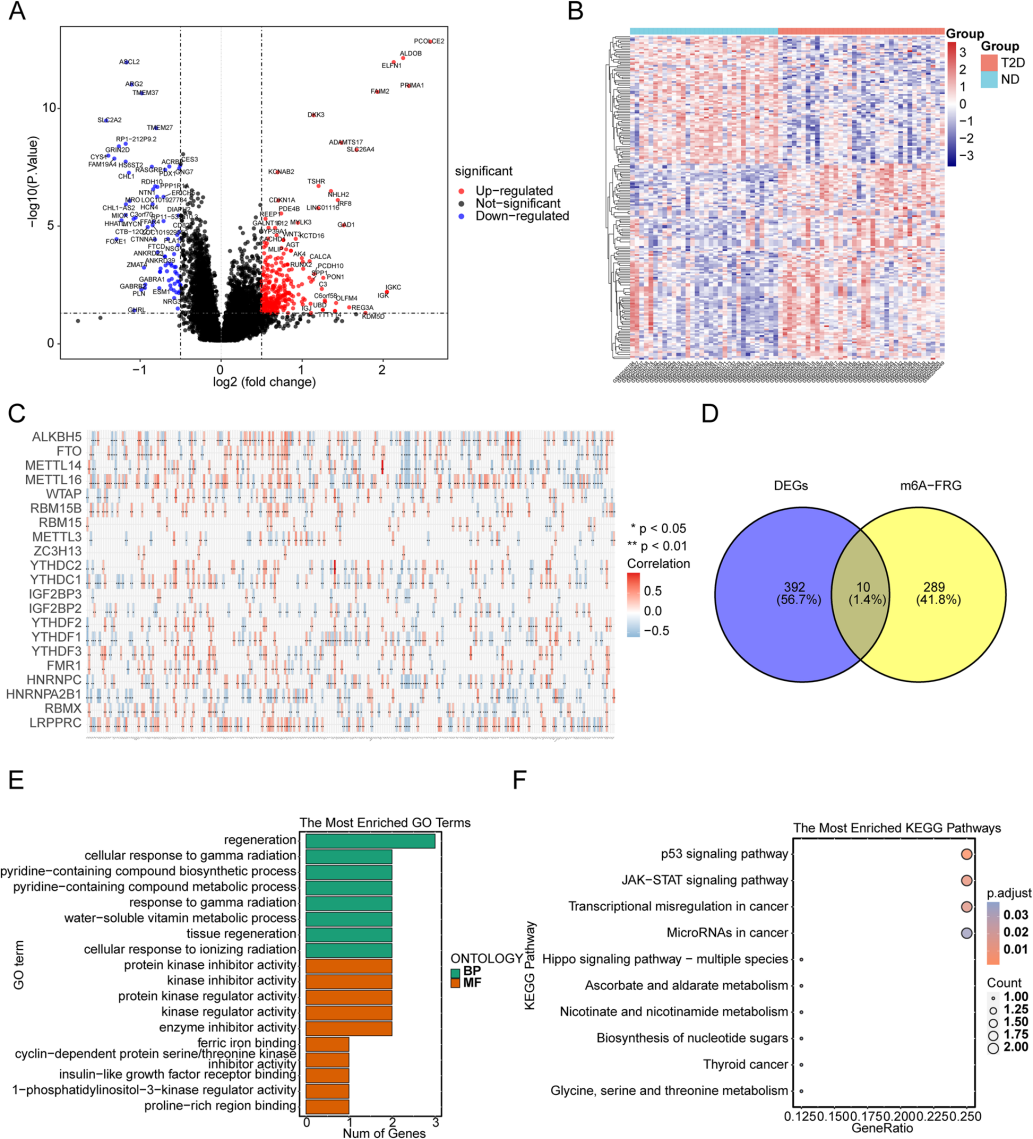
Identification of 10 differentially expressed m6A-FRGs (DEm6A-FRGs) in Gene Expression Omnibus (GEO) datasets. **A** Volcano plot showed 324 up-regulated (red) and 78 down-regulated genes (blue) in two clusters (|log_2_FC|> 0.5, adj.P-value < 0.05). **B** Heatmap of the correlation between T2D group and ND group. **C** Heatmap of the correlation between m6A-related genes and FRGs. *p < 0.05, **p < 0.01. **D** Venn diagram for differentially expressed genes (DEGs) and m6A-related ferroptosis genes (m6A-FRGs). **E** The column plot of the 8 Gene Ontology (GO) Biological Process (BP) terms and top 10 GO Molecular Function (MF) terms were enriched for 10 DEm6A-FRGs. **F** The bubble plot of the top 10 Kyoto Encyclopedia of Genes and Genomes (KEGG) pathways were enriched for 10 DEm6A-FRGs

There were 402 DEGs (324 up-regulated and 78 down-regulated) between 36 T2DM and 32 HC samples (Fig. 1A&B, Supplement Table 1). Then the correlations between 23 m6A methylation regulatory-related genes and 621 ferroptosis-related genes were calculated, and totals of 299 m6A-FRGs were obtained (Fig. 1C, Supplement Table 2). Finally, 10 DEm6A-FRGs (MIOX, MYCN, ARHGEF26-AS1, CD82, ADAM23, YAP1, SOCS1, CDKN1A, NNMT and PSAT1) were obtained by intersecting 402 DEGs and 299 m6A-FRGs (Fig. 1D).

Besides, these genes were enriched to 8 Gene Ontology (GO) biological processes and 17 GO molecular functions, including regeneration, pyridine-containing compound biosynthetic and metabolic process, water-soluble vitamin metabolic process, protein kinase inhibitor activity, and etc. In addition, these genes were enriched to 13 Kyoto Encyclopedia of Genes and Genomes (KEGG) pathways, including p53 signaling pathway, JAK-STAT signaling pathway, type II diabetes mellitus, and etc. (Fig. 1E&F, Supplement Table 3&4).

### Construction and evaluation of the diagnostic model of T2DM with 4 biomarkers

**Fig. 2:**
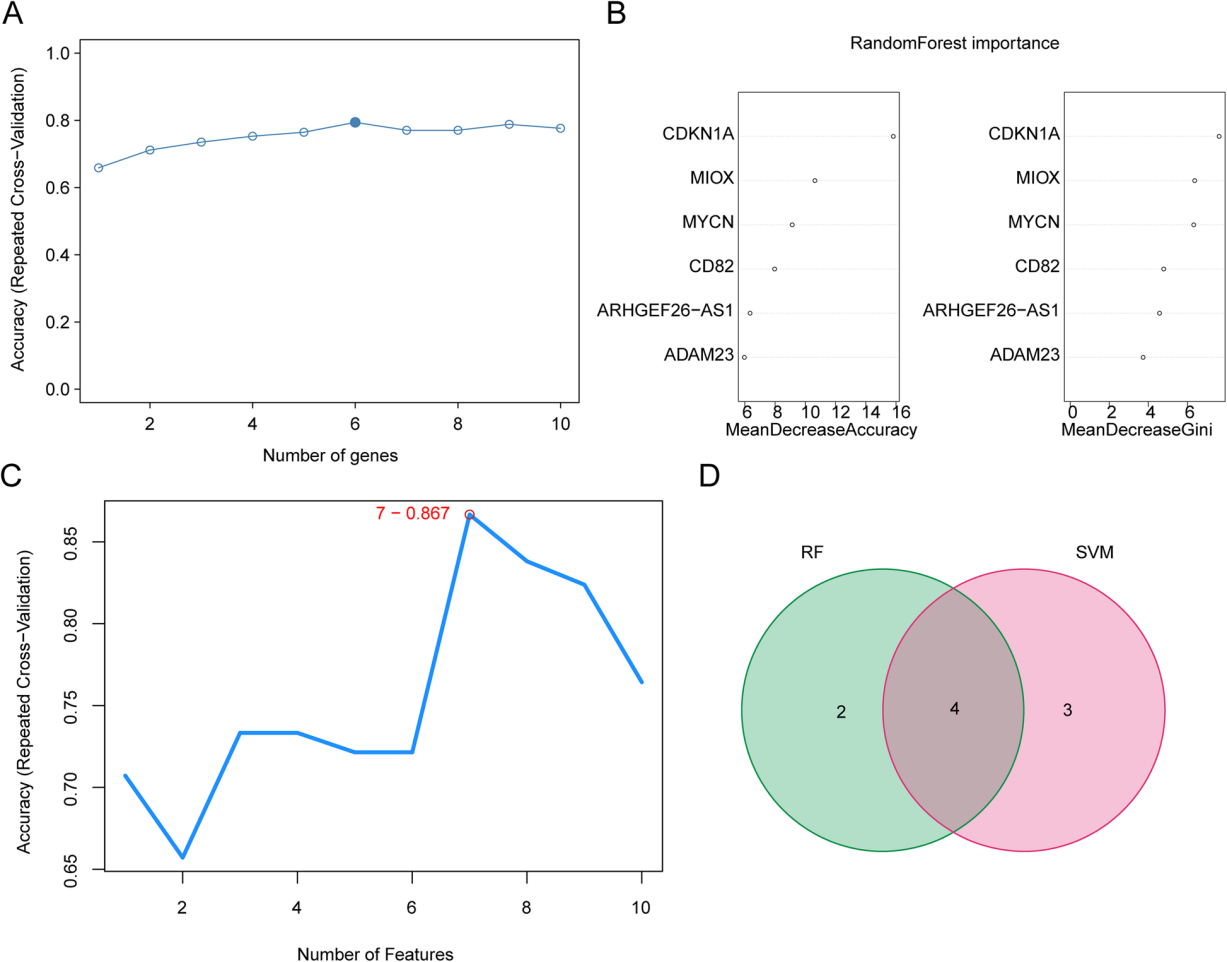
Screening of 4 biomarkers by Random Forest (RF) method and support vector machine recursive feature elimination (SVM-RFE) method. **A-B** 6 characterized genes were screened from 10 DEm6A-FRGs by RF method. **C** 7 characterized genes were screened from 10 DEm6A-FRGs by SVM-RFE method. **D** Venn diagram of characterized genes

Six characteristic genes, including CDKN1A, MIOX, MYCN, CD82, ARHGEF26-AS1 and ADAM23, were screened by RF method, and 7 feature genes, including CDKN1A, MYCN, CD82, ARHGEF26-AS1, MIOX, YAP1 and NNMT were screened by SVM-RFE method (Fig. 2A&B). Then, 4 common genes, including CDKN1A, MIOX, MYCN and CD82 were regarded as the biomarkers for further analysis (Fig. 2C, D).

**Fig. 3:**
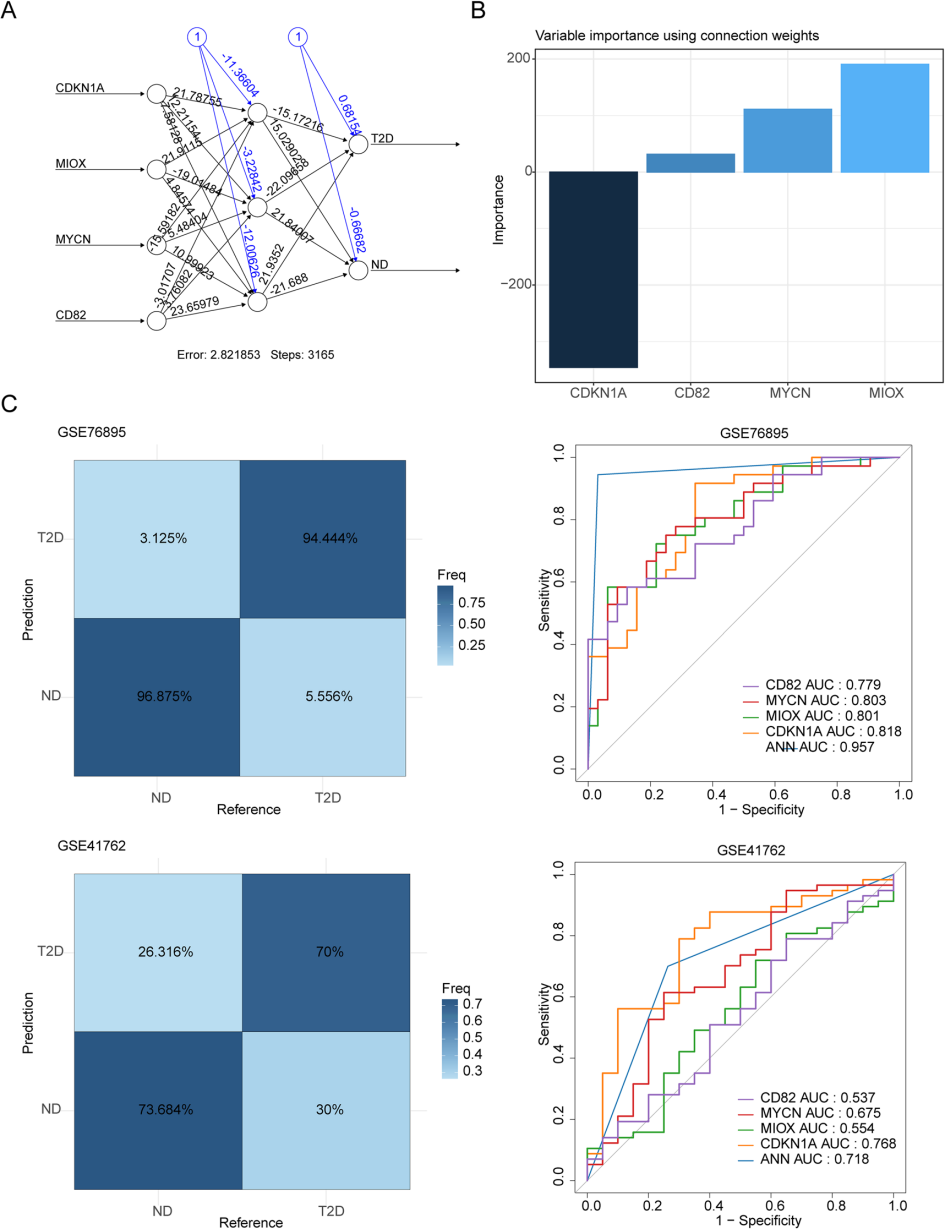
Construction and validation of an Artificial Neural Network (ANN) model based on 4 biomarkers. **A** Visualization of the ANN model. The black arrows and associated numbers are weights, which you can think of as the variable's contribution to the next node's the degree of contribution of that variable to the next node. The blue line is the bias weight. **B** Visualization of the importance of the independent variables on the predicted results of the model. **C** Confusion matrix and operating characteristic curves (ROC) curve for hub genes in GSE76895 and GSE41762 datasets

**Fig. 4:**
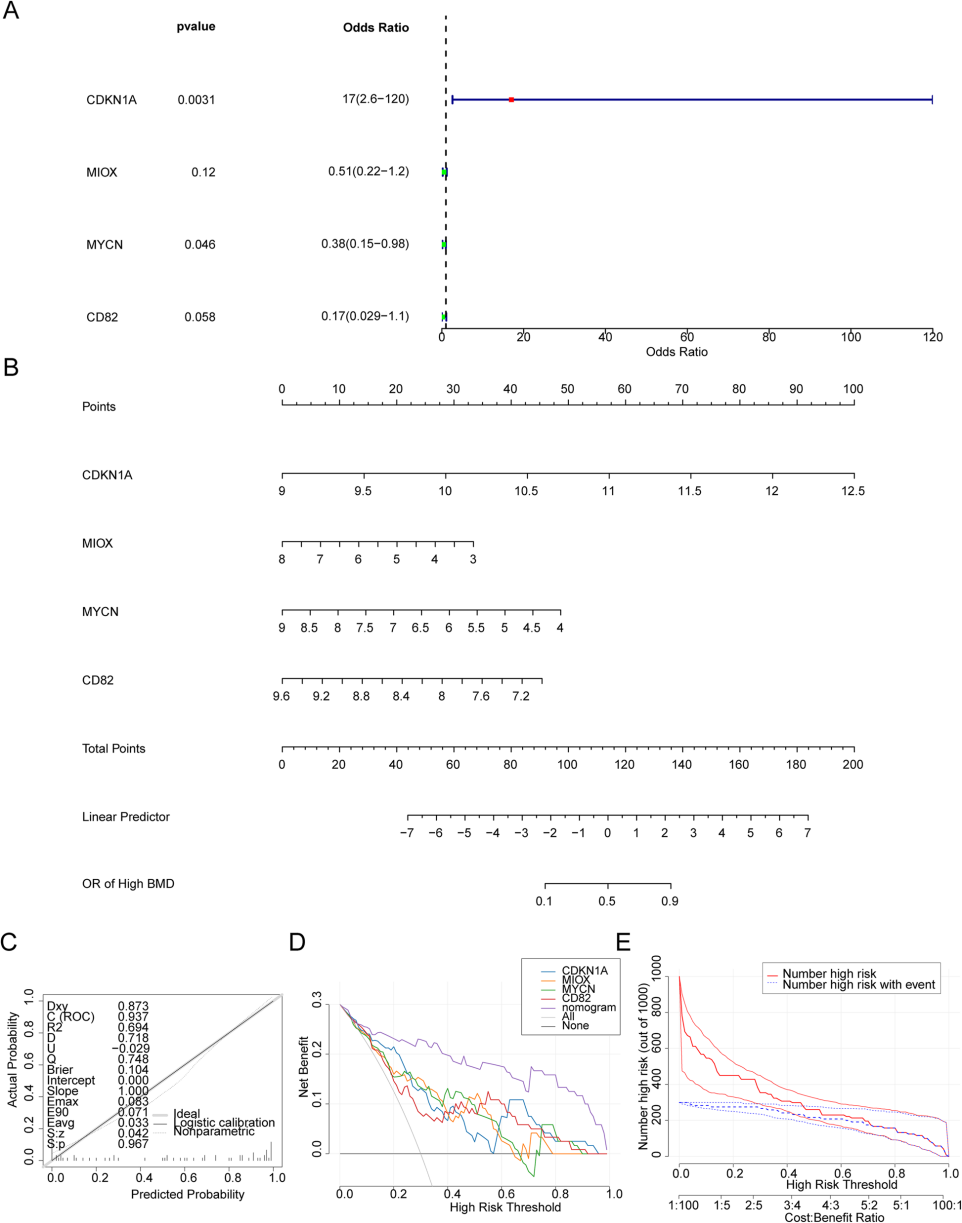
Construction and validation of logistic regression models for biomarkers. **A** Forest plot of the multivariable Cox analyses of the biomarkers. **B** Nomogram model of four biomarkers in the GSE76895 dataset. **C** Calibration curves of the nomogram for predicting the Odd Ratio of T2DM. The 45-degree line represents the ideal prediction. **D** Decision curve analyses (DCA) curve of the nomogram. **E** Clinical impact curves predicted with the nomogram. The number high risk indicates the number of people classified as high risk by the simple model at each threshold, and the number high risk outcome is the number of true positives at each threshold

Based on it, the ANN model and the nomogram with these 4 biomarkers were constructed, respectively. In ANN model, CDKN1A was the most important biomarker for forecasting T2DM (Fig. 3A,B). Moreover, the areas under ROC (AUC values) of ANN model were great than 0.7 in both GSE76895 and GSE41762 (Fig. 3B), these results showed that the ANN model could be used as an effective diagnostic model. On the other hand, LRM showed the similar results, which CDKN1A was the most important biomarker for forecasting T2DM (odd ratio = 17) (Fig. 4A). The nomogram with these 4 biomarkers were constructed, the calibration curve of the nomogram showed that the slope was the closest to 1 and ROC was 0.937, which indicated that the prediction model could be used as an effective diagnostic model (Fig. 4B, C). The results of the DCA were showed that the benefit rate of the nomogram model was higher than individual biomarker (Fig. 4D), and the results of clinical impact curve were showed that the “Number high risk” curve almost coincided with the “Number high risk with event” curve at the benefit ratio threshold from 0 to 1 (Fig. 4E), all of these indicating that the nomogram model has accurate predictive power.

### The GSEA of biomarkers

**Fig. 5:**
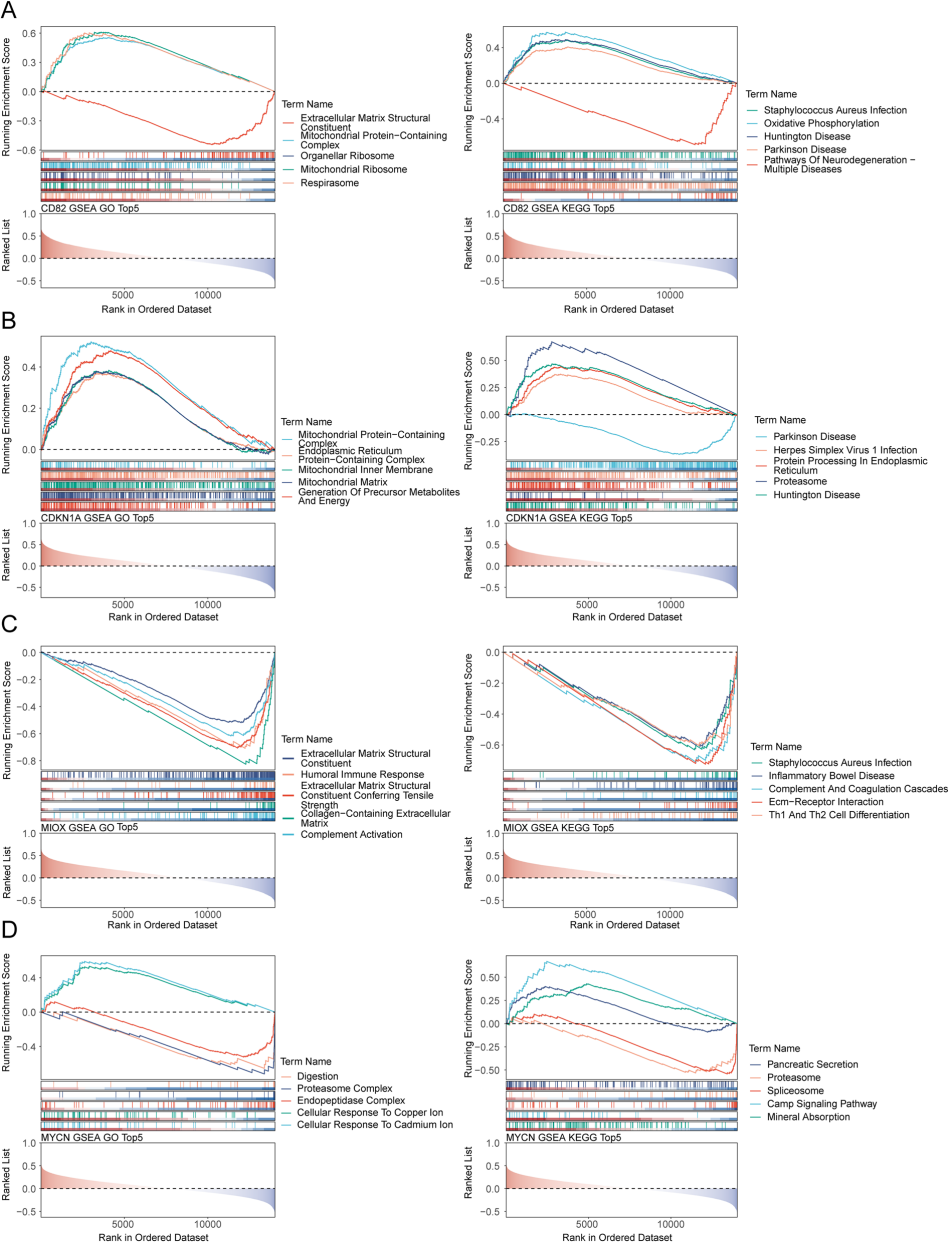
Gene Set Enrichment Analysis (GSEA) analysis of 4 biomarkers in GO terms and KEGG pathways (|NES|> 1, adj. *P*-value < 0.05, Q–value < 0.2). **A-D** GSEA analysis of CD82 (**A**), CDKN1A (**B**), MIOX (**C**) and MYCN (**D**) in GO terms and KEGG pathways

The results of GSEA of 4 biomarkers were shown in the Fig. 5A-D. The results revealed that CD82 was associated with the function of respirasome, CDKN1A was associated with the function of generation of precursor metabolites and energy, MIOX was associated with the function of humoral immune response extracellular matrix structural, MYCN was associated with the function of digestion, proteasome complex, endopeptidase complex, cellular response to copper ion and cadmium ion. Noticeable, the function of extracellular matrix structural constituent lowly expressed in both CD82 and MIOX, the function of mitochondrial protein-containing complex highly expressed in both CD82 and CDKN1A. On the other hands, the pathway of Staphylococcus Aureus infection highly enriched in CD82 and MIOX, the pathway of proteasome highly enriched in CDKN1A and MYCN. In addition, the pathway of huntington disease and parkinson disease highly expressed in CD82 and CDKN1A.

### Molecular mechanism analysis of 4 biomarkers

**Fig. 6:**
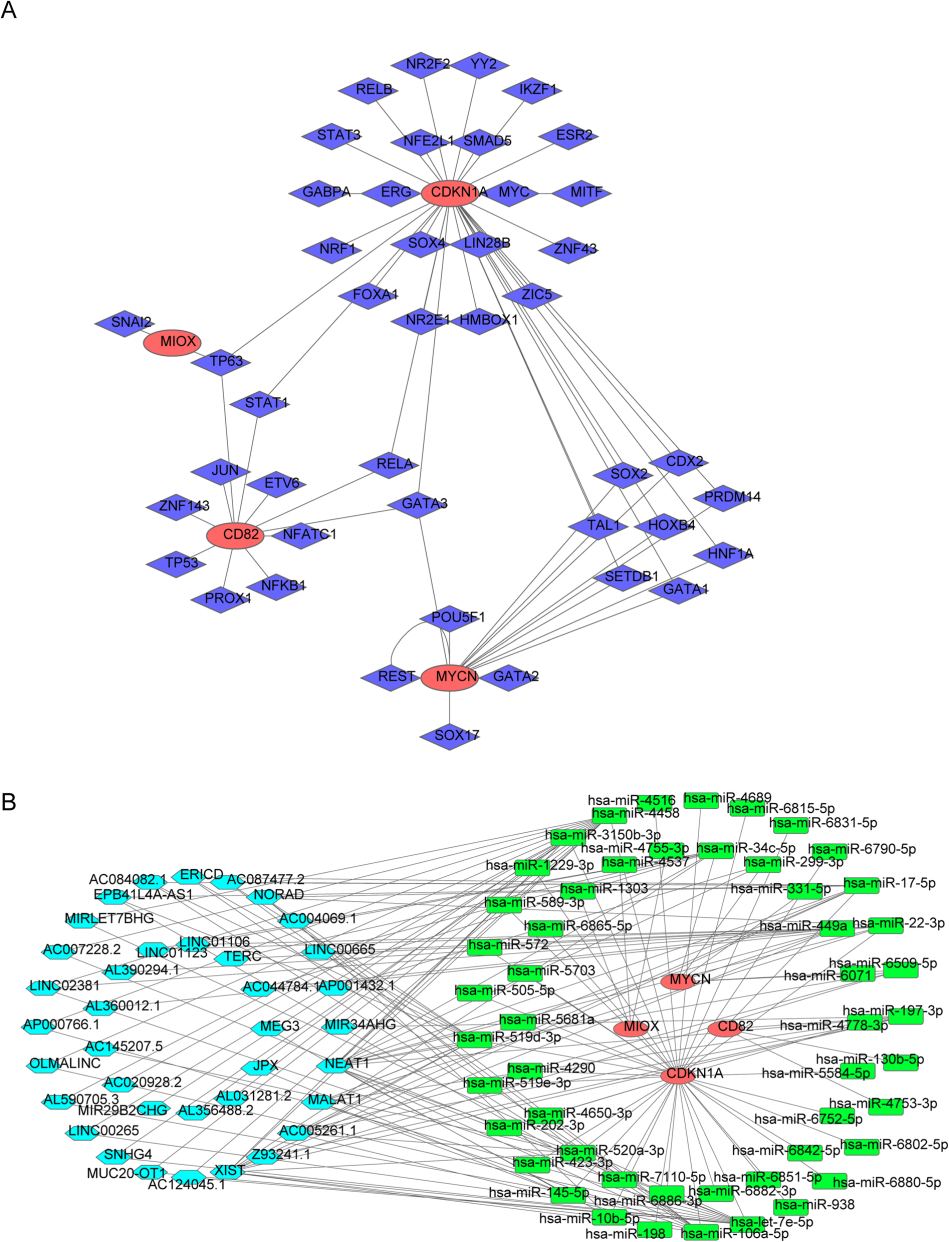
Molecular mechanism analysis of 4 biomarkers. **A** Construction of transcription factors (TF)-mRNA regulatory network. Blue diamonds represent transcription factors, and orange ovals represent biomarkers. **B** Construction of competing endogenous RNAs (ceRNA) network. Orange ovals represent biomarkers, azure hexagons represent lncRNAs, and green rectangular shapes represent miRNAs

A total of 44 TFs associated with biomarkers were downloaded and 59 TF-mRNA relationship pairs were obtained for constructing the TF-mRNA regulatory network (Fig. 6A, Table 1, Supplement Table 5). In this network, TP63 was key TF which could regulate CD82, CDKN1A and MIOX at the same time, GATA3 was key TF which could regulate CD82, CDKN1A and MYCN at the same time, STAT1 and RELA were the common TFs of CD82 and CDKN1A, besides, CDX2, PRDM14, GATA1, HNF1A, HOXB4, SOX2, TAL1 and SETDB1 were the common TFs of CDKN1A and MYCN.

**Table 1:** Number of predicted transcription factors (TFs) and intersection TFs per database

Overlapping_Genes	ChEA3_Number	KnockTF_Number	Overlap_Number
CD82	116	168	11
CDKN1A	511	174	32
MIOX	17	32	2
MYCN	151	119	14

A total of 51 targeted miRNAs were obtained, which includes 3 targeted miRNAs of CD82, 39 targeted miRNAs of CDKN1A, 3 targeted miRNAs of MIOX, and 6 targeted miRNAs of MYCN. Based on it, 37 lncRNAs were predicted for further studies (Table 2, Supplement Table 6). Moreover, the ceRNA network was constructed, which contains 4 biomarkers, 51 miRNAs and 37 lncRNAs (Fig. 6B, Supplement Table 7). In this network, we found that there were 12 common lncRNAs between has-miR-4458 and has-let-7e-5p, 9 common lncRNAs among has-miR-17-5p, has-miR-519d-3p and has-miR-106a-5p. It is worth noting that XIST was a key lncRNA that associated with 12 targeted miRNAs, which could influence CDKN1A.

**Table 2:** Number of miRNAs screened for 4 biomarkers by miRWalk database

genesymbol	miRWalk_Number	MiRTarBase_Number	Overlap_Number
CD82	9	13	3
CDKN1A	347	137	39
MIOX	3	39	3
MYCN	11	46	6

### Drug prediction

**Fig. 7:**
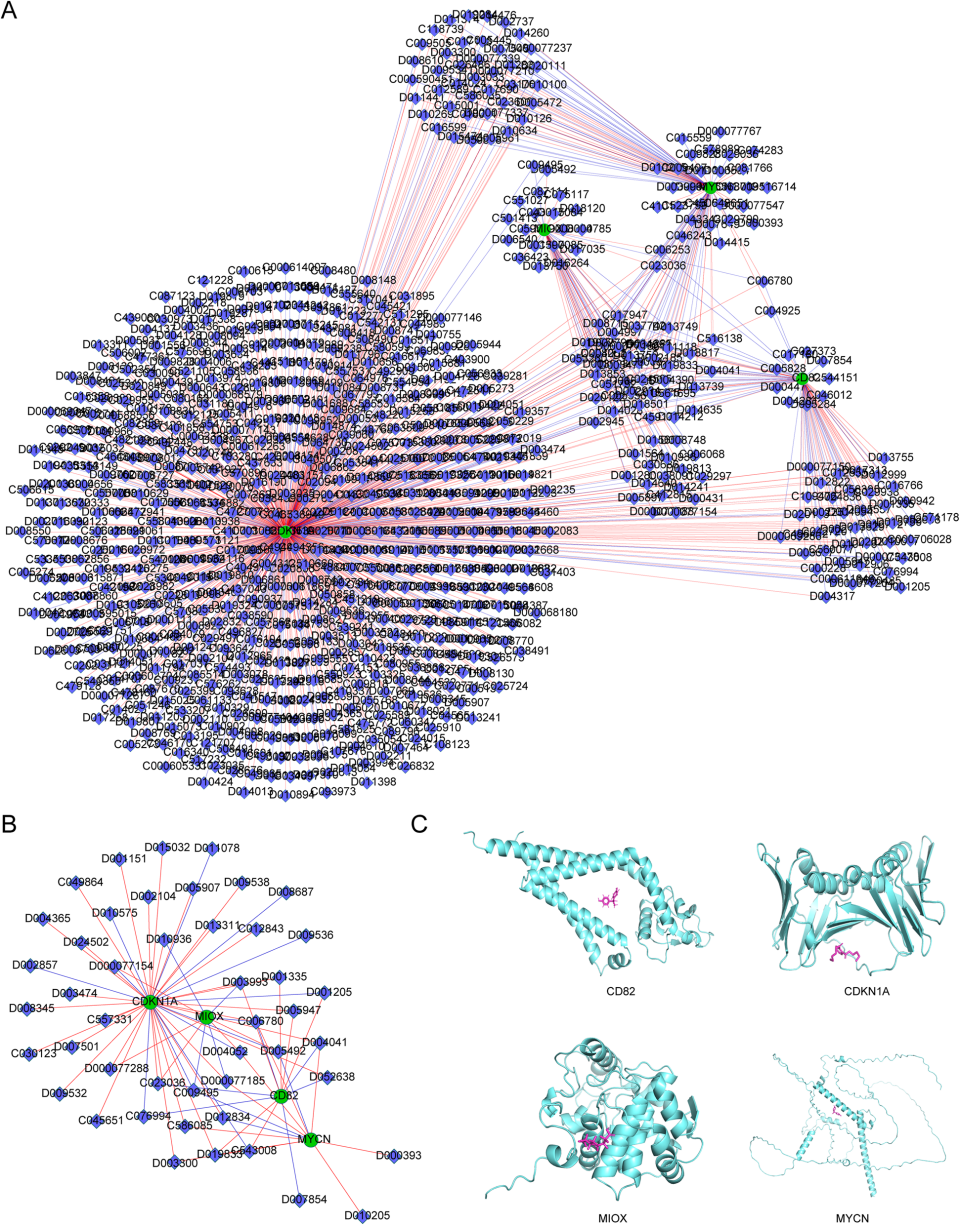
Biomarker-related drug prediction. **A** Drug prediction for 4 biomarkers. Green circles are biomarkers, blue diamonds are drug small molecules, red edges are up-regulated expression regulation, blue edges are down-regulated expression regulation. **B** The drug networks of biomarkers in disease. **C** The Molecular docking results of four biomarkers with key drugs

The results of interaction relationship between drugs and 4 biomarkers were showed that totals of 804 drugs and 966 relationship pairs were predicted, among them, 46 drugs were associated with T2DM. It is worth noting that C006780 could increase the expression of CDKN1A, CD82, MYCN and decrease the expression of MIOX at the same time. D019833 could increase the expression of CDKN1A, CD82 and MYCN, C009495 could decrease the expression of CDKN1A, MIOX and MYCN. C023036 could increase the expression of CDKN1A, MIOX, and decrease the expression of CD82. D000077185 could increase the expression of CDKN1A, MYCN, and decrease the expression of MIOX. D004052 could increase the expression of MYCN, MIOX, and decrease the expression of CDKN1A. D005492 could increase the expression of MIOX, and decrease the expression of CDKN1A, MYCN. D003993 could increase the expression of CDKN1A, CD82, and decrease the expression of MIOX. D004041 could increase the expression of MYCN, MIOX, and decrease the expression of CD82 (Fig. 7A, B, Table 3). Besides, the interaction scores and networks between T2DM and the biomarkers were showed in Table 4.

**Table 3:** Interaction scores of T2D with biomarkers

Gene Symbol	Gene ID	Disease Name	Disease ID	Direct Evidence	Inference Network	Inference Score	Reference Count	
CDKN1A	1026	Diabetes Mellitus, Type 2	MESH:D003924		Air Pollutants|alpha-Tocopherol|Arsenic|Ascorbic Acid|Berberine|bis(4-hydroxyphenyl)sulfone|bisphenol A|Cadmium|Chromium|cinnamaldehyde|Copper|coumarin|Curcumin|daidzein|Dibutyl Phthalate|Dietary Fats|Diethylnitrosamine|Drugs, Chinese Herbal|epigallocatechin gallate|Folic Acid|Genistein|ginsenoside Re|Gliclazide|Glucose|Glutathione|Hemin|Irbesartan|Iron|Lead|Manganese|Metformin|Niacinamide|Nickel|Nicotine|Orlistat|Particulate Matter|perfluorooctane sulfonic acid|perfluorooctanoic acid|perfluoroundecanoic acid|Pesticides|Pioglitazone|Plant Extracts|Polychlorinated Biphenyls|Resveratrol|Rosiglitazone|Silver|Streptozocin|TAK-875|titanium dioxide|Tolbutamide|Troglitazone|Vehicle Emissions|Zinc	77.81	151	
MIOX	55,586	Diabetes Mellitus, Type 2	MESH:D003924		bisphenol A|Dibutyl Phthalate|Diethylnitrosamine|Folic Acid|Iron|perfluorooctanoic acid|Plant Extracts|Resveratrol|Rosiglitazone|titanium dioxide|Troglitazone|Vehicle Emissions	34.34	55	
MYCN	4613	Diabetes Mellitus, Type 2	MESH:D003924		Air Pollutants|bisphenol A|Copper|coumarin|Dietary Fats|Diethylnitrosamine|Folic Acid|Genistein|Pantothenic Acid|perfluoroundecanoic acid|Resveratrol|Silver|titanium dioxide	28.46	38	
CD82	3732	Diabetes Mellitus, Type 2	MESH:D003924		Ascorbic Acid|bis(4-hydroxyphenyl)sulfone|bisphenol A|Dibutyl Phthalate|Dietary Fats|Genistein|Glucose|Lead|Particulate Matter|perfluorooctane sulfonic acid|perfluorooctanoic acid|Vehicle Emissions	29.47	33

**Table 4:** Interaction scores and networks between Type 2 diabetes mellitus (T2DM) and biomarkers

Chemical Name	Chemical ID	CAS RN	Gene Symbol	Gene ID	Interaction	Interaction Actions	Reference Count	Organism Count	
bisphenol A	C006780	########	CD82	3732	bisphenol A results in decreased expression of CD82 mRNA	decreases^expression	4	2	
Arsenic	D001151	7440–38-2	CDKN1A	1026	Arsenic results in increased expression of CDKN1A mRNA	increases^expression	2	1	
Cadmium	D002104	7440–43-9	CDKN1A	1026	[Cadmium Chloride results in increased abundance of Cadmium] which results in increased expression of CDKN1A mRNA	increases^expression	2	1	
Dibutyl Phthalate	D003993	84–74-2	CDKN1A	1026	Dibutyl Phthalate results in increased expression of CDKN1A mRNA	increases^expression	2	2	
Drugs, Chinese Herbal	D004365		CDKN1A	1026	Drugs, Chinese Herbal results in increased expression of CDKN1A mRNA	increases^expression	2	1	
Silver	D012834	7440–22-4	CDKN1A	1026	Silver analog results in increased expression of CDKN1A mRNA	increases^expression	2	1	
titanium dioxide	C009495	13,463–67-7	CDKN1A	1026	titanium dioxide results in decreased expression of CDKN1A mRNA	decreases^expression	2	2	
Dibutyl Phthalate	D003993	84–74-2	MIOX	55,586	Dibutyl Phthalate results in decreased expression of MIOX mRNA	decreases^expression	2	1	
perfluorooctanoic acid	C023036	335–67-1	MIOX	55,586	perfluorooctanoic acid results in increased expression of MIOX mRNA	increases^expression	2	2	
alpha-Tocopherol	D024502	########	CDKN1A	1026	alpha-Tocopherol inhibits the reaction [Acrylonitrile results in increased expression of CDKN1A mRNA]	increases^expression	1	1	
Ascorbic Acid	D001205	50–81-7	CDKN1A	1026	[Ascorbic Acid co-treated with Zinc Acetate co-treated with motexafin gadolinium] results in decreased expression of CDKN1A mRNA	decreases^expression	1	1	
bis(4-hydroxyphenyl)sulfone	C543008	########	CDKN1A	1026	bis(4-hydroxyphenyl)sulfone results in decreased expression of CDKN1A mRNA	decreases^expression	1	1	
bisphenol A	C006780	########	CDKN1A	1026	[bisphenol A co-treated with cylindrospermopsin] results in increased expression of CDKN1A mRNA	increases^expression	1	1	
Chromium	D002857	7440–47-3	CDKN1A	1026	Chromium results in decreased expression of CDKN1A mRNA	decreases^expression	1	1	
cinnamaldehyde	C012843		CDKN1A	1026	cinnamaldehyde results in increased expression of CDKN1A mRNA	increases^expression	1	1	
Copper	D003300	7440–50-8	CDKN1A	1026	[Chelating Agents binds to Copper] which results in increased expression of CDKN1A mRNA	increases^expression	1	1	
coumarin	C030123	91–64-5	CDKN1A	1026	coumarin analog results in increased expression of CDKN1A mRNA	increases^expression	1	1	
Curcumin	D003474	458–37-7	CDKN1A	1026	[Curcumin binds to and results in increased activity of VDR protein] which results in increased expression of CDKN1A mRNA	increases^expression	1	1	
Dietary Fats	D004041		CDKN1A	1026	[Dietary Fats co-treated with Choline deficiency] results in increased expression of CDKN1A mRNA	increases^expression	1	1	
Diethylnitrosamine	D004052	55–18-5	CDKN1A	1026	AHR protein inhibits the reaction [Diethylnitrosamine results in decreased expression of CDKN1A mRNA]	decreases^expression	1	1	
epigallocatechin gallate	C045651	989–51-5	CDKN1A	1026	[epigallocatechin gallate co-treated with leptomycin B] results in increased expression of CDKN1A mRNA	increases^expression	1	1	
Folic Acid	D005492	59–30-3	CDKN1A	1026	[1,2-Dimethylhydrazine co-treated with Folic Acid] results in decreased expression of CDKN1A mRNA	decreases^expression	1	1	
Genistein	D019833	446–72-0	CDKN1A	1026	[Calcitriol co-treated with Genistein] results in increased expression of CDKN1A mRNA	increases^expression	1	1	
ginsenoside Re	C049864	52,286–59-6	CDKN1A	1026	ginsenoside Re results in increased expression of CDKN1A mRNA	increases^expression	1	1	
Gliclazide	D005907	21,187–98-4	CDKN1A	1026	Gliclazide inhibits the reaction [Hydrogen Peroxide results in increased expression of CDKN1A mRNA]	increases^expression	1	1	
Glucose	D005947	50–99-7	CDKN1A	1026	[Glucose co-treated with Deferoxamine] results in increased expression of CDKN1A mRNA	increases^expression	1	1	
Iron	D007501	7439–89-6	CDKN1A	1026	[Nickel co-treated with Iron co-treated with Tungsten] results in increased expression of CDKN1A mRNA	increases^expression	1	1	
Manganese	D008345	7439–96-5	CDKN1A	1026	Manganese deficiency results in increased expression of CDKN1A mRNA	increases^expression	1	1	
Metformin	D008687	657–24-9	CDKN1A	1026	Metformin inhibits the reaction [Testosterone results in decreased expression of CDKN1A mRNA]	decreases^expression	1	1	
Niacinamide	D009536	98–92-0	CDKN1A	1026	[Butyric Acid co-treated with Niacinamide co-treated with Glucaric Acid] inhibits the reaction [9,10-Dimethyl-1,2-benzanthracene results in decreased expression of CDKN1A mRNA]	decreases^expression	1	1	
Nickel	D009532	7440–02-0	CDKN1A	1026	[Nickel co-treated with Cobalt co-treated with Tungsten] results in increased expression of CDKN1A mRNA	increases^expression	1	1	
Nicotine	D009538		CDKN1A	1026	Acetylcysteine inhibits the reaction [Nicotine results in increased expression of CDKN1A mRNA]	increases^expression	1	1	
Particulate Matter	D052638		CDKN1A	1026	4-((3-bromophenyl)amino)-6,7-dimethoxyquinazoline inhibits the reaction [Particulate Matter results in increased expression of CDKN1A mRNA]	increases^expression	1	1	
perfluorooctane sulfonic acid	C076994	1763–23-1	CDKN1A	1026	perfluorooctane sulfonic acid results in decreased expression of CDKN1A mRNA	decreases^expression	1	1	
perfluorooctanoic acid	C023036	335–67-1	CDKN1A	1026	perfluorooctanoic acid inhibits the reaction [Calcitriol results in increased expression of CDKN1A mRNA]	increases^expression	1	1	
perfluoroundecanoic acid	C586085		CDKN1A	1026	perfluoroundecanoic acid results in increased expression of CDKN1A mRNA	increases^expression	1	1	
Pesticides	D010575		CDKN1A	1026	Pesticides results in decreased expression of CDKN1A mRNA	decreases^expression	1	1	
Plant Extracts	D010936		CDKN1A	1026	[Plant Extracts co-treated with Glucaric Acid] inhibits the reaction [9,10-Dimethyl-1,2-benzanthracene results in increased expression of CDKN1A mRNA]	increases^expression	1	1	
Polychlorinated Biphenyls	D011078		CDKN1A	1026	Polychlorinated Biphenyls analog results in decreased expression of CDKN1A mRNA	decreases^expression	1	1	
Resveratrol	D000077185		CDKN1A	1026	2-(2-amino-3-methoxyphenyl)-4H-1-benzopyran-4-one inhibits the reaction [Resveratrol results in increased expression of CDKN1A mRNA]	increases^expression	1	1	
Rosiglitazone	D000077154		CDKN1A	1026	Rosiglitazone results in decreased expression of CDKN1A mRNA	decreases^expression	1	1	
Streptozocin	D013311	18,883–66-4	CDKN1A	1026	2-amino-6-boronohexanoic acid inhibits the reaction [Streptozocin results in increased expression of CDKN1A mRNA]	increases^expression	1	1	
TAK-875	C557331		CDKN1A	1026	TAK-875 results in increased expression of CDKN1A mRNA	increases^expression	1	1	
Troglitazone	D000077288	97,322–87-7	CDKN1A	1026	[Troglitazone co-treated with IFNG protein] results in increased expression of CDKN1A mRNA	increases^expression	1	1	
Vehicle Emissions	D001335		CDKN1A	1026	4-((3-bromophenyl)amino)-6,7-dimethoxyquinazoline inhibits the reaction [Vehicle Emissions results in increased expression of CDKN1A mRNA]	increases^expression	1	1	
Zinc	D015032	7440–66-6	CDKN1A	1026	[PCI 5002 co-treated with Zinc] results in increased expression of CDKN1A mRNA	increases^expression	1	1	
bisphenol A	C006780	########	MIOX	55,586	bisphenol A results in decreased expression of MIOX mRNA	decreases^expression	1	1	
Diethylnitrosamine	D004052	55–18-5	MIOX	55,586	Diethylnitrosamine results in increased expression of MIOX mRNA	increases^expression	1	1	
Folic Acid	D005492	59–30-3	MIOX	55,586	[Methionine deficiency co-treated with Choline deficiency co-treated with Folic Acid deficiency] results in increased expression of MIOX mRNA	increases^expression	1	1	
Plant Extracts	D010936		MIOX	55,586	[Plant Extracts co-treated with Resveratrol] results in decreased expression of MIOX mRNA	decreases^expression	1	1	
Resveratrol	D000077185		MIOX	55,586	[Plant Extracts co-treated with Resveratrol] results in decreased expression of MIOX mRNA	decreases^expression	1	1	
Rosiglitazone	D000077154		MIOX	55,586	Rosiglitazone results in increased expression of MIOX mRNA	increases^expression	1	1	
titanium dioxide	C009495	13,463–67-7	MIOX	55,586	[titanium dioxide co-treated with Azoxymethane co-treated with Dextran Sulfate] results in decreased expression of MIOX mRNA	decreases^expression	1	1	
Troglitazone	D000077288	97,322–87-7	MIOX	55,586	Troglitazone results in increased expression of MIOX mRNA	increases^expression	1	1	
Air Pollutants	D000393		MYCN	4613	[Air Pollutants results in increased abundance of [Ozone co-treated with Soot]] which results in increased expression of MYCN mRNA	increases^expression	1	1	
bisphenol A	C006780	########	MYCN	4613	bisphenol A results in increased expression of MYCN mRNA	increases^expression	1	1	
Copper	D003300	7440–50-8	MYCN	4613	[Copper co-treated with Chlorpyrifos] results in increased expression of MYCN mRNA	increases^expression	1	1	
Dietary Fats	D004041		MYCN	4613	Dietary Fats results in increased expression of MYCN mRNA	increases^expression	1	1	
Diethylnitrosamine	D004052	55–18-5	MYCN	4613	[Diethylnitrosamine co-treated with Phenobarbital] results in increased expression of MYCN mRNA	increases^expression	1	1	
Folic Acid	D005492	59–30-3	MYCN	4613	[Methionine deficiency co-treated with Choline deficiency co-treated with Folic Acid deficiency] results in decreased expression of MYCN mRNA	decreases^expression	1	1	
Genistein	D019833	446–72-0	MYCN	4613	Genistein results in increased expression of MYCN mRNA	increases^expression	1	1	
Pantothenic Acid	D010205	79–83-4	MYCN	4613	Pantothenic Acid results in increased expression of MYCN mRNA	increases^expression	1	1	
perfluoroundecanoic acid	C586085		MYCN	4613	perfluoroundecanoic acid results in increased expression of MYCN mRNA	increases^expression	1	1	
Resveratrol	D000077185		MYCN	4613	Resveratrol inhibits the reaction [Dietary Fats results in increased expression of MYCN mRNA]	increases^expression	1	1	
Silver	D012834	7440–22-4	MYCN	4613	Silver results in decreased expression of MYCN mRNA	decreases^expression	1	1	
titanium dioxide	C009495	13,463–67-7	MYCN	4613	titanium dioxide results in decreased expression of MYCN mRNA	decreases^expression	1	1	
Ascorbic Acid	D001205	50–81-7	CD82	3732	Ascorbic Acid results in increased expression of CD82 mRNA	increases^expression	1	1	
bis(4-hydroxyphenyl)sulfone	C543008	########	CD82	3732	bis(4-hydroxyphenyl)sulfone results in increased expression of CD82 mRNA	increases^expression	1	1	
Dibutyl Phthalate	D003993	84–74-2	CD82	3732	Dibutyl Phthalate results in increased expression of CD82 mRNA	increases^expression	1	1	
Dietary Fats	D004041		CD82	3732	Dietary Fats results in decreased expression of CD82 mRNA	decreases^expression	1	1	
Genistein	D019833	446–72-0	CD82	3732	Genistein results in increased expression of CD82 mRNA	increases^expression	1	1	
Glucose	D005947	50–99-7	CD82	3732	[INS protein co-treated with Glucose] results in increased expression of CD82 mRNA	increases^expression	1	0	
Lead	D007854	7439–92-1	CD82	3732	Lead results in decreased expression of CD82 mRNA	decreases^expression	1	1	
Particulate Matter	D052638		CD82	3732	Particulate Matter results in decreased expression of CD82 mRNA	decreases^expression	1	1	
perfluorooctane sulfonic acid	C076994	1763–23-1	CD82	3732	perfluorooctane sulfonic acid results in decreased expression of CD82 mRNA	decreases^expression	1	1	
perfluorooctanoic acid	C023036	335–67-1	CD82	3732	perfluorooctanoic acid results in decreased expression of CD82 mRNA	decreases^expression	1	1	
Vehicle Emissions	D001335		CD82	3732	[Allergens co-treated with Vehicle Emissions] results in increased expression of CD82 mRNA	increases^expression	1	1	

The results of molecular docking between 4 biomarkers and key drugs were showed in Fig. 7C. The docking affinity between CD82 and bisphenol A was -7.5 kcal/mol. The docking affinity between CDKN1A and Dibutyl Phthalate was -5.0 kcal/mol. The docking affinity between MIOX and perfluorooctanoic acid was -7.1 kcal/mol. The docking affinity between MYCN and bisphenol A was -6.2 kcal/mol. All of these results suggested a good combination of biomarkers and targeted drugs.

**Fig. 8:**
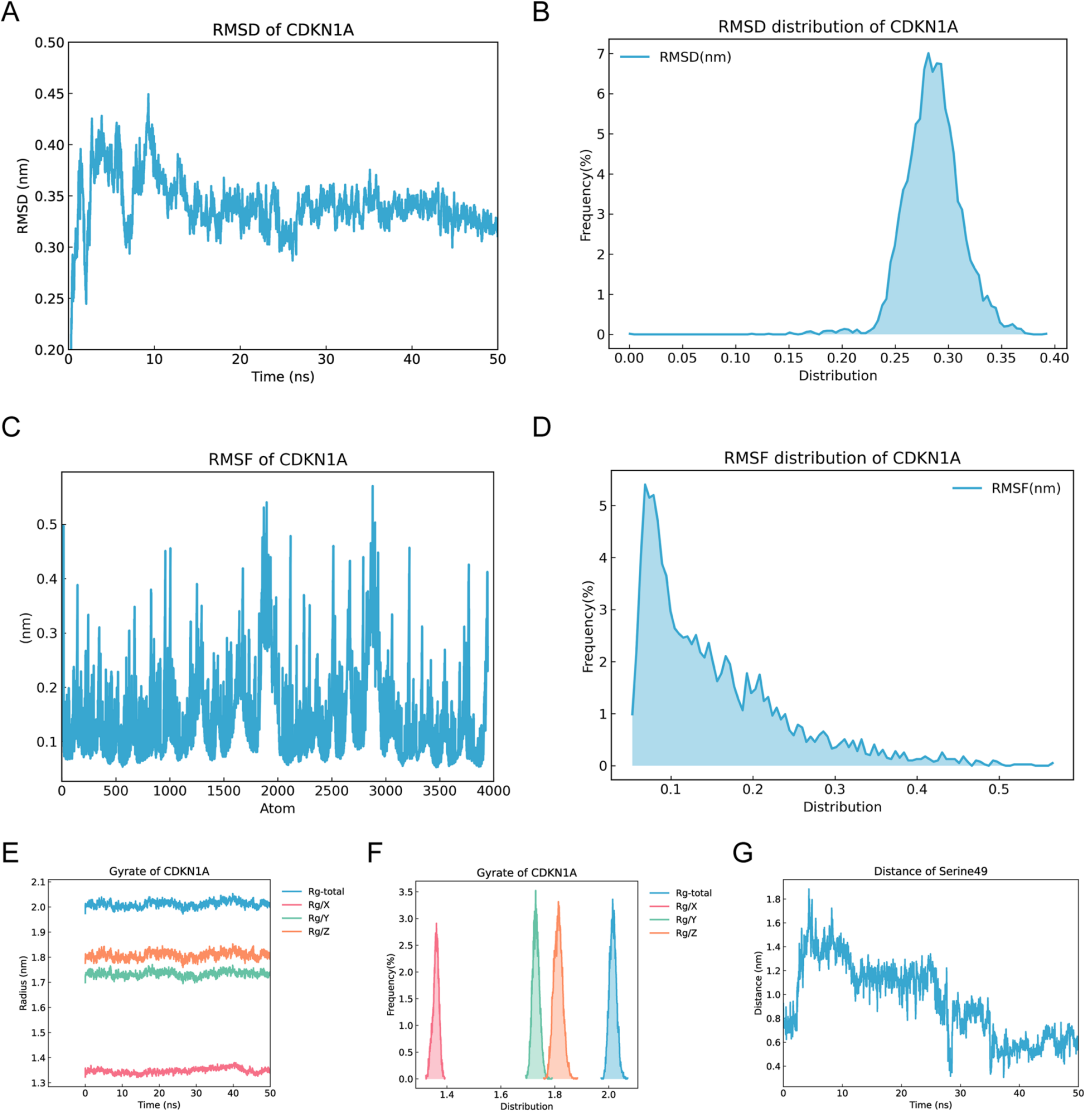
MDs of CDKN1A and Dibutyl Phthalate. **A-B** RMSD curve of CDKN1A protein. **C-D** RMSF plot of amino acid flexibility and exercise intensity of CDKN1A protein. **E–F** RoG plot of CDKN1A protein. **G** Dynamic distance changes of key amino acids and ligands

To further validate the reliability of molecular docking, MDs were implemented in this study, where root mean square deviation (RMSD), root mean square fluctuation (RMSF) and radius of gyration (RoG) were included in the simulation analysis. The results of this study were presented only for CDKN1A, and the results of MDs for the remaining biomarkers were shown in Supplementary Figs. 1–3. RMSD curves can reveal the positional changes of the protein between the conformation and the initial conformation during the simulation. The RMSD curve fluctuated in the early stages due to the interaction between the complex and the solvent. After that, the protein was in a steady state at 40 ns to 50 ns, and the RMSD value falls between 0.25 and 0.35, indicating that the binding of CDKN1A and dibutyl phthalate was relatively stable (Fig. 8A-B). The RMSF is the average of the atomic position change for time, which can characterize the flexibility and movement intensity of protein amino acids throughout the simulation. The results showed that the protein amino acids were flexible and bind stably to the small molecule drug ligands within the simulation duration (Fig. 8C-D). RoG can characterize the tightness of the protein structure, and similarly can be relied upon to characterize the changes in the degree of peptide chain looseness of the proteins during the simulation. As can be seen in the Fig. 8E-F, CDKN1A-dibutyl phthalate has a stable RoG, which was consistent with the RMSD curve results, indicating a more stable binding. Furthermore, in the simulation process of protein and small molecule ligand, the distance between key amino acids and ligand showed a dynamic change trend. The change of the distance between Dibutyl phthalate and the active site of Serine amino acid residue No. 49 of CDKN1A ligand protein tended to stabilize around 50 ns, and the fluctuation was mainly distributed around 0.6, which could be considered that the binding of dibutyl phthalate to the active site of CDKN1 was stable (Fig. 8G).

### Expression verification of biomarkers

**Fig. 9:**
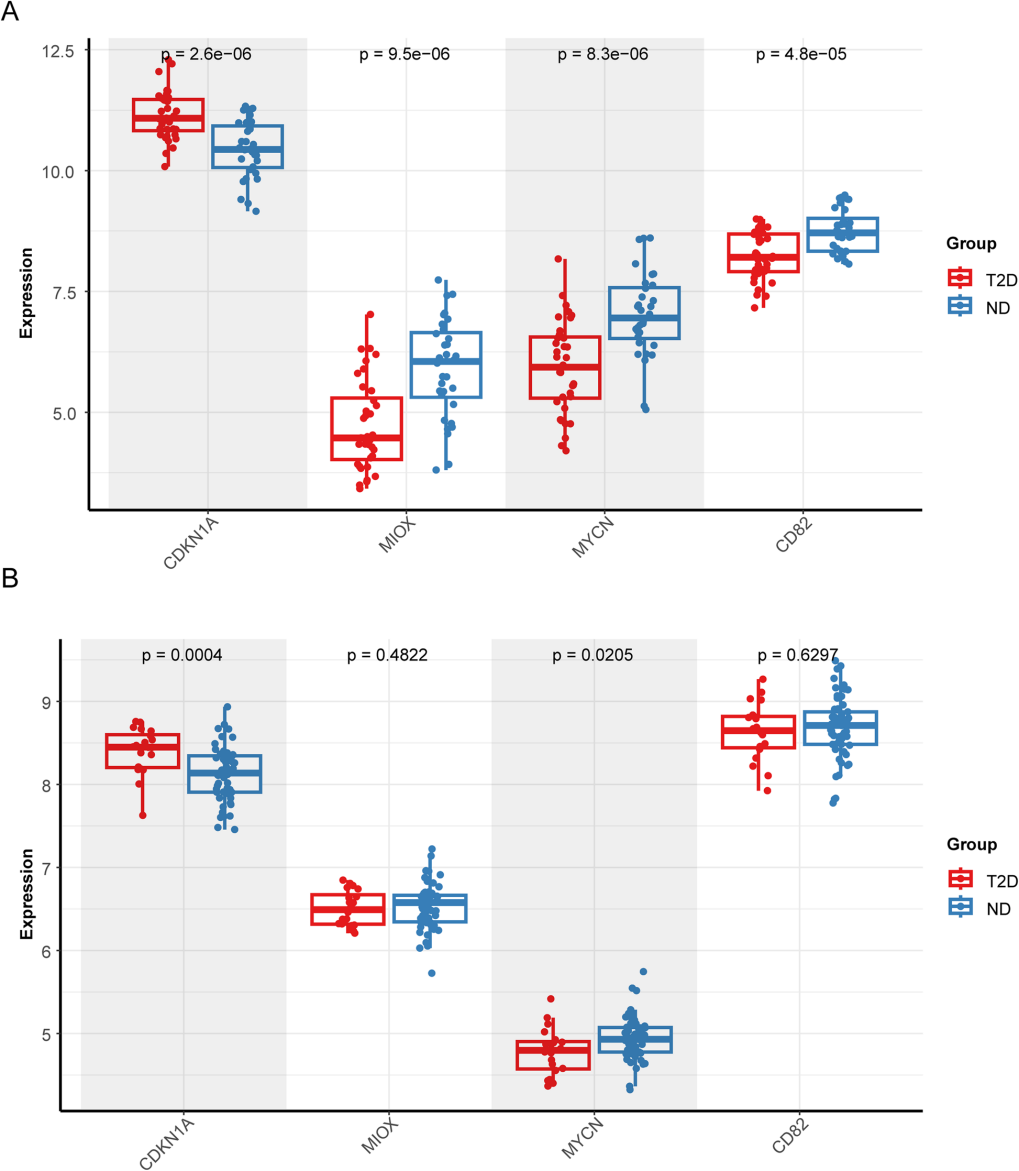
Expression verification of biomarkers. **A** The gene expression level in GSE76895 dataset. **B** The gene expression level in GSE41762 dataset

The expression of 4 biomarkers between the T2DM and HC samples were compared, and the results showed that MIOX, MYCN, CD82 were lowly expressed, and CDKN1A was highly expressed in T2DM samples, among them, the expression of CDKN1A and MYCN showed significantly different in both of GSE76895 and GSE41762 (Fig. 9A, B).

### The alternation of protein expression in primary pancreatic islet cells (CP-R015) under hyperglycemia conditions

**Fig. 10:**
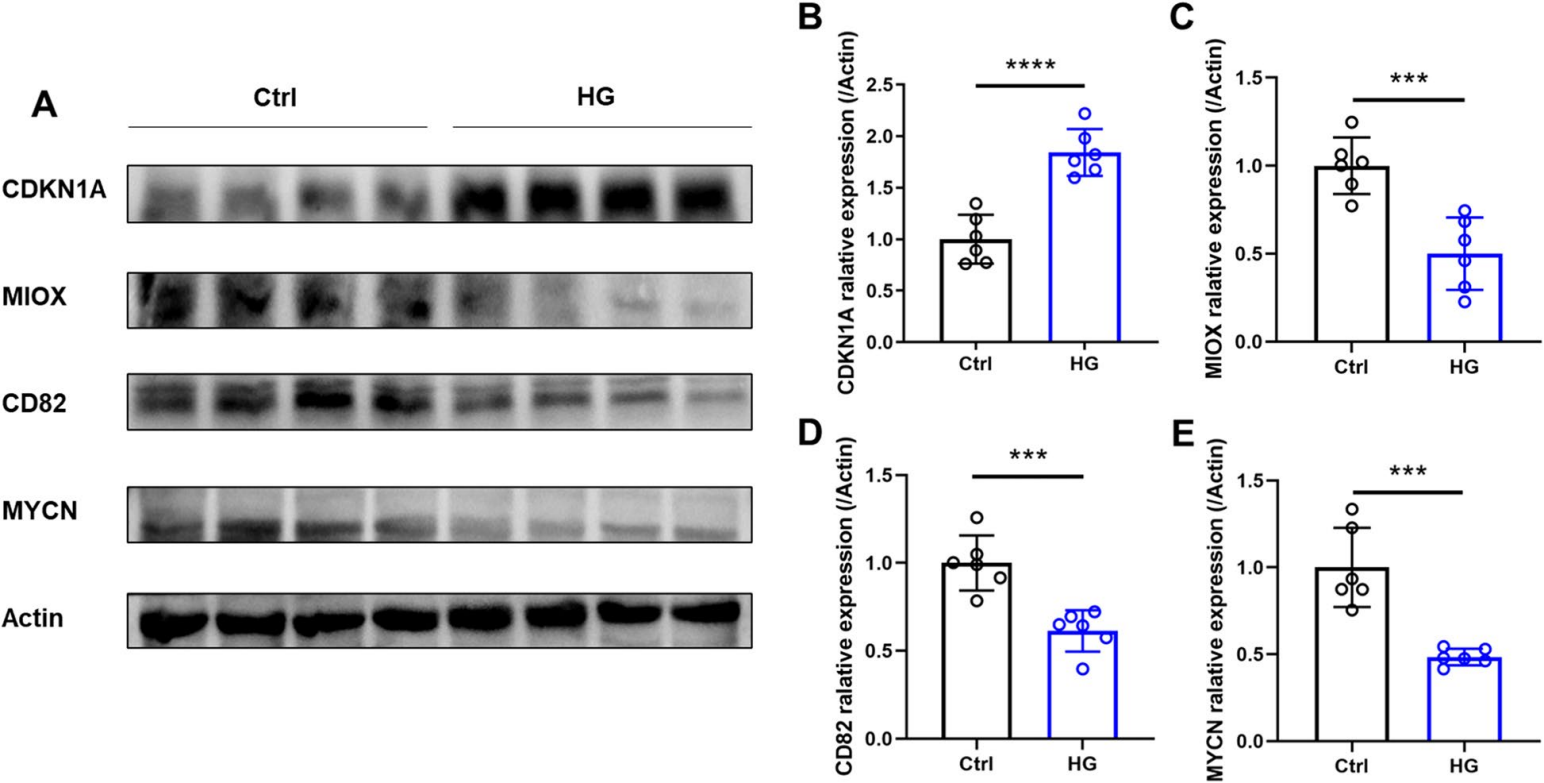
Protein expression of the biomarkers. **A** Western blotting results of CDKN1A, MIOX,CD82 and MYCN. **B-E** The column plot of changes in protein expression of CDKN1A (**B**), MIOX (**C**), CD82 (**D**), and MYCN (**E**). ***p < 0.001, ****p < 0.0001

To investigate the expression of 4 biomarkers in cells, we cultured primary pancreatic islet cells (CP-R015) using both regular and high glucose medium, and then performed protein blotting. As shown in the Fig. 10A-E, compared with the control group (Ctrl), the high glucose group (HG) showed a significant increase in CDKN1A protein expression, while the expression of MIOX, CD82, and MYCN proteins was significantly reduced.

## Discussion

T2DM is a common chronic wasting disease, which is characterized by insulin resistance and insulin secretion deficiency leading to dysglycemia [24]. Iron ions are cofactors of various enzymes and participate in various biosynthetic pathways in the body [25]. Imbalance of iron metabolism is a key factor in the development of endocrine diseases. Abnormal iron metabolism in the body leads to various pathological and physiological changes, such as ferroptosis. Ferroptosis damages to pancreatic beta cells, which leads to a decrease in insulin secretion and induces insulin resistance in the liver, fat, and muscles [26]. The m6A is one of the most common RNA modifications in eukaryotes, and m6A induces ferroptosis by regulating the autophagy signaling pathway [5]. At present, only a few studies have explored the relationship between m6A and ferroptosis in T2DM. Also the specific mechanism of m6A's post transcriptional regulation of ferroptosis is unknown. The study of the key genes of m6A regulating ferroptosis may provide effective therapeutic targets and diagnostic markers for T2DM. Through the analysis of 36 T2DM and 32 healthy controls in dataset GSE76895, the ferroptosis related genes regulated by m6A, which are differentially expressed in T2DM, were determined. Through the comprehensive analysis of random forest (RF) and support vector machine recursive feature elimination (SVM-RFE), four characteristic genes were identified, namely: CDKN1A, MIOX, MYCN and CD82. Then, Artificial neural network (ANN) model was constructed for these four feature genes, and the diagnostic efficacy of these four feature genes and ANN model was tested. We found that these four genes had good efficacy for T2DM diagnosis in the training set GSE76895. In the validation set GSE41762, although the diagnostic efficacy of the four genes decreased, the ANN model could still improve the diagnostic efficacy.

CDKN1A is a cycle dependent kinase inhibitor -1 that mediates cell proliferation, differentiation, and apoptosis by regulating the expression of cyclin [27, 28]. Researches have shown that CDKN1A involved in regulating the p53 signaling pathway to regulate cell differentiation and apoptosis. When CDKN1A is activated by p53 transcription, it upregulates and prevents the release of cell cycle regulatory factors, leading to cell cycle arrest and an increase in pancreatic beta cells apoptosis [29]. In addition, overexpression of p21/CDKN1A controls cell cycle entry into G1 phase, reducing pancreatic function, pancreatic beta cells proliferation and pancreatic beta cells insulin secretion [30]. Our data demonstrated that CDKN1A is highly expressed in T2DM, which is consistent with previous study [31]. In a related study, it was found that the degree of CDKN1A methylation was reduced in the islets of diabetic patients compared to normal subjects, which promoted the enhancement of CDKN1A expression. At the same time, the researchers found that overexpression of CDKN1A in cloned beta cells led to impaired insulin secretion, and overexpression of CDKN1A in cloned alpha cells led to increased glucagon secretion [32]. Therefore, CDKN1A causes T2DM by regulating the cell cycle, affecting insulin secretion and glucagon secretion, and how to promote CDKN1A methylation will become the key to the treatment of T2DM.

CD82 is a transfer inhibitory factor that is a member of the tetraspanin membrane protein family. It interacts with chemokines and integrins, controlling related signaling pathways to regulate cell motility, adhesion, migration, aging, and apoptosis. It has been found that CD82^+^ cells in human islets can secrete insulin more effectively than CD82^−^ cells, and CD82 can promote the differentiation of endocrine progenitor cells into mature beta cells [33]. In our study, we found a decrease in CD82 in the islets of T2DM patients, which may be related to the enhancement of CD82 methylation, which inhibits its expression.

MYCN is a transcription factor whose main biological effect is to regulate the expression of multiple genes during cell proliferation, growth, aging, metabolism, differentiation, and apoptosis [34]. In a study on the growth cycle of mouse pancreatic islet beta cells, it was found that MYCN is an upstream mediator for pancreatic beta cell expansion, and regulates pancreatic cell growth by mediating the expansion cycle and differentiation of pancreatic beta cells [35]. The overexpression of MYCN makes mouse beta cells enter G1 cell cycle, which is the growth and differentiation stagnation, leading to the growth and differentiation obstacle of beta cells and diabetes [36]. Signe Horn et al. found that MYCN is not only involved in regulating cell growth, survival, metabolism, and death, but also in maintaining cellular redox balance [37, 38]. Overexpression of MYCN promotes the upregulation of TFRC and mediates GPX4 pathway dependent ferroptosis [39]. Meanwhile, overexpression of MYCN increases intracellular iron load and unstable iron pool (LIP) levels, ultimately leading to enhanced lipid peroxidation and ferroptosis [37]. At the same time, the strong proliferative signal overexpressed by MYCN often leads to apoptosis of pancreatic islet beta cells [40]. In the study of neuroblastoma, it was found that MYCN amplification is related to the methylation status of its binding site, and the degree of methylation is negatively correlated with the degree of gene expression [41]. The low expression of MYCN in T2DM may be due to a protective mechanism of the body under hyperglycemia. Ferroptosis, cell apoptosis and methylation play an important role in the progress of T2DM, and MYCN has the co-function of regulating cell proliferation, growth, differentiation and other aspects. Through the biological effects of MYCN, corresponding research will be carried out to clarify new ideas for the treatment of diabetes based on the mechanism of MYCN on methylation and ferroptosis.

Myo-Inositol oxygenase (MIOX) is a non heme ferritin whose transcription is regulated by oxidative stress, free fatty acids, and high glucose environments [42]. In diabetes, the expression of MIOX depends on the demethylation of the MIOX promoter under hyperglycemia [43]. MIOX overexpression promotes the production of reactive oxygen species (ROS), which can oxidize proteins, lipids, carbohydrates and DNA, leading to mitochondrial DNA damage and cell dysfunction [44]. Due to the upregulation of MIOX in T2DM, oxidative stress and endoplasmic reticulum stress are exacerbated, leading to mitochondrial energy metabolism disorders and exacerbating pancreatic islet beta cells damage [45]. MIOX can also regulate inflammatory damage by activating NLRP3 inflammasomes and altering the release of inflammatory mediators [46]. In the state of hyperglycemia, abnormal expression of MIOX activates NLRP3 inflammasomes, leading to increased release of inflammatory mediators, promoting inflammatory response, leading to pancreatic cell damage, and exacerbating insulin resistance. Research has shown that overexpression of MIOX promotes pancreatic islet beta cells ferroptosis, which may be due to the overexpression of MIOX leading to lipid peroxidation and a decrease in GPX4 activity and NADPH levels [47]. NADPH levels are sensitive to ferroptosis and help eliminate lipid hydroperoxides [48]. GPX4 is a key antioxidant enzyme, Ferroptosis can be blocked by inhibiting lipid hydroperoxides that reduce glutathione (GSH) [49]. When MIOX is overexpressed, on the one hand, it accelerates ferroptosis in pancreatic islet cells by downregulating GPX4 activity and intracellular GSH concentration, and on the other hand, it promotes ferroptosis and leads to insulin resistance by regulating intracellular levels of iron [49, 50]. In view of the role that MIOX plays in tissues, it can improve the oxidative stress, inflammatory reaction and ferroptosis of cells by regulating the expression level of MIOX, thus improving the blood sugar level of diabetes and the complications of diabetes.

Our study is the first to identify the key m6A regulated ferroptosis gene in T2DM through bioinformatics and machine learning methods, and conduct experimental validation. However, there are also certain shortcomings in our research. Firstly, the experimental subjects were only at the cellular level and did not undergo in vivo experiments. We will conduct further research in the following studies. In addition, the impact of four key m6A regulated ferroptosis related genes in T2DM and their biological mechanisms need to be further explored to improve their corresponding mechanisms.

## Conclusion

In summary, our study integrated literature-reported m6A regulatory genes, ferroptosis related genes from the FerrDb database, and T2DM transcriptome data from the GEO database to identify m6A ferroptosis genes. We then used machine learning algorithms to identify four biomarkers of T2DM. Additionally, we constructed TF-mRNA regulatory and ceRNA networks of these biomarkers to predict potential drugs, which can provide potential targets for clinical diagnosis of T2DM and a theoretical basis for further understanding of the driving mechanism of T2DM. This study revealed the potential molecular mechanisms of m6A-related ferroptosis genes in T2DM, which could provide novel insights for the clinical diagnosis and treatment of T2DM.

## Supplementary Information

Supplementary Material 1: Supplement Table 1 Differentially expressed genes between T2D groups and HC groups.

Supplementary Material 2: Supplement Table 2 Ferroptosis genes significantly associated with N6-methyladenosine (m6A)-regulated genes.

Supplementary Material 3: Supplement Table 3 The 8 Gene Ontology (GO), Biological Process (BP) terms, and 17 GO Molecular Function (MF) terms were enriched for 10 differentially expressed m6A-FRGs (DEm6A-FRGs).

Supplementary Material 4: Supplement Table 4 The 13 Kyoto Encyclopedia of Genes and Genomes (KEGG) pathways were enriched for 10 DEm6A-FRGs.

Supplementary Material 5: Supplement Table 5 Construction of 59 pairs of TF-mRNAs in the TF-mRNA regulatory network.

Supplementary Material 6: Supplement Table 6 Construction of 150 pairs of regulatory relationships in the competing endogenous RNAs (ceRNA) regulatory network.

Supplementary Material 7: Supplement Table 7 Construction of ceRNA network with 4 biomarkers, 51 miRNAs, and 37 lncRNAs.

Supplementary Material 8: Figure S1 MDs of CD82 and bisphenol A. (A-B) RMSD curve of CD82 protein. (C-D) RMSF plot of amino acid flexibility and exercise intensity of CD82 protein. (E–F) RoG plot of CD82 protein. (G) dynamic distance changes of key amino acids and ligands

Supplementary Material 9: Figure S2 MDs of MIOX and perfluorooctanoic acid (A-B) RMSD curve of MIOX protein. (C-D) RMSF plot of amino acid flexibility and exercise intensity of MIOX protein. (E–F) RoG plot of MIOX protein. (G) dynamic distance changes of key amino acids and ligands

Supplementary Material 10: Figure S3 MDs of MYCN and bisphenol A. (A-B) RMSD curve of MYCN protein. (C-D) RMSF plot of amino acid flexibility and exercise intensity of MYCN protein. (E–F) MYCN protein RoG plot. (G) dynamic distance changes of key amino acids and ligands

Supplementary Material 11

## Data Availability

No datasets were generated or analysed during the current study.

## Abbreviations

m6A: N6-methyladenosine
T2DM: Type 2 diabetes mellitus
GEO: Gene Expression Omnibus
DEGs: Differentially expressed genes
m6A-FRGs: M6A-related ferroptosis genes
DEm6A-FRGs: Differentially expressed m6A-FRGs
ANN: Artificial neural network
RF: Random forest
SVM-RFE: Support vector machine recursive feature elimination
TF: Transcription factors
ceRNA: Competing endogenous RNAs
GSEA: Gene Set Enrichment Analysis
HC: Healthy controls
ROC: Receiver operating characteristic
LRM: Logistic regression model
DCA: Decision curve
PVDF: Polyvinylidene difluoride membranes
GO: Gene Ontology
KEGG: Kyoto Encyclopedia of Genes and Genomes
Ctrl: Control group
HG: High glucose group
WBC: White blood cells
LIP: Load and unstable iron pool
MIOX: Myo-Inositol oxygenase
ROS: Reactive oxygen species
GSH: Glutathione
