# Supplementary figures and images for: Identification of biomarkers and mechanism exploration of ferroptosis related genes regulated by m6A in type 2 diabetes mellitus
Source: Hereditas. 2025 Feb 18;162:24. doi: 10.1186/s41065-025-00385-9 (PMC11834627; doi:10.1186/s41065-025-00385-9)

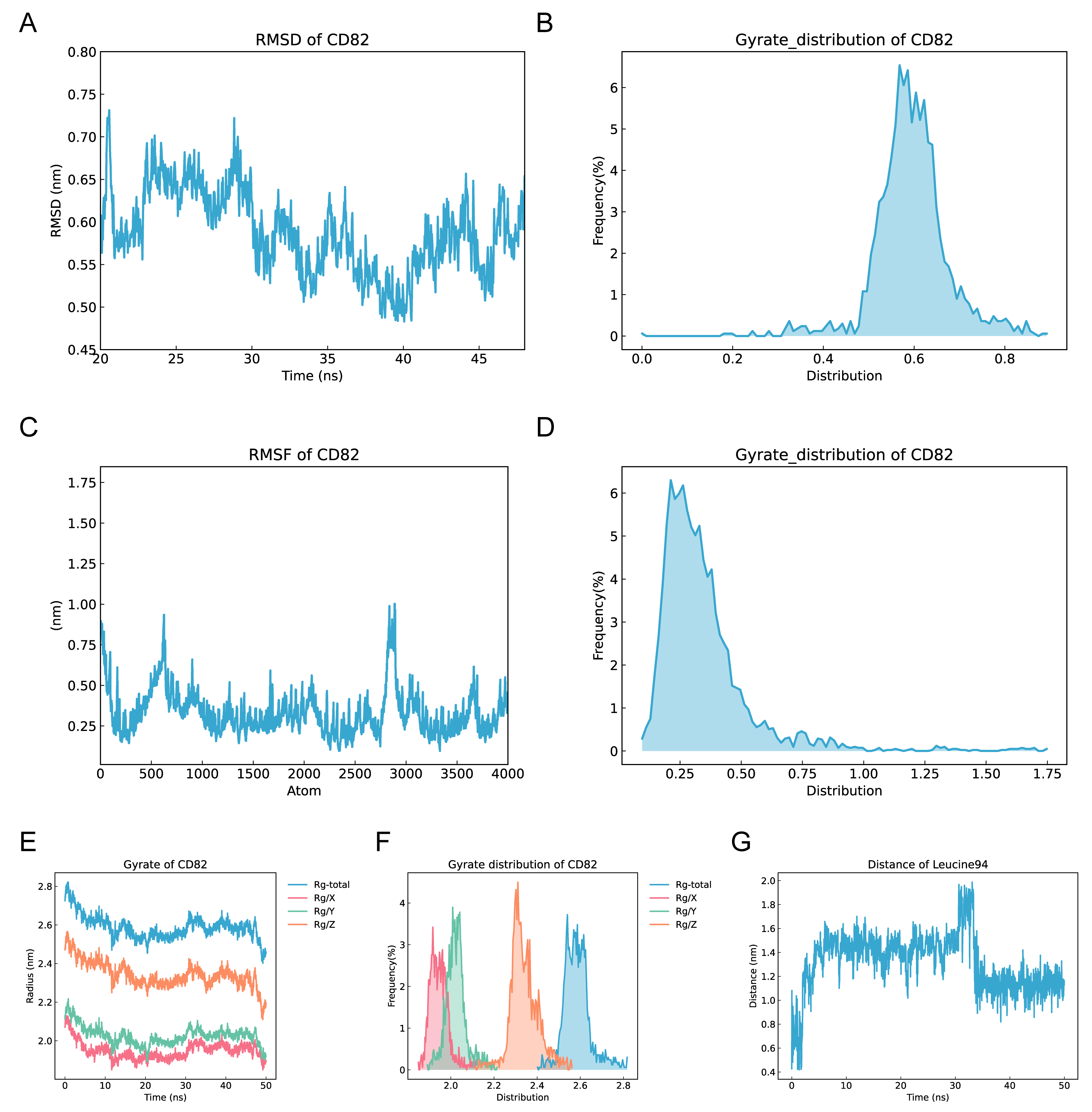

Supplement: Supplementary file 8 — Supplementary Material 8: Figure S1 MDs of CD82 and bisphenol A. (A-B) RMSD curve of CD82 protein. (C-D) RMSF plot of amino acid flexibility and exercise intensity of CD82 protein. (E–F) RoG plot of CD82 protein. (G) dynamic distance changes of key amino acids and ligands [file 41065_2025_385_MOESM8_ESM.tif]

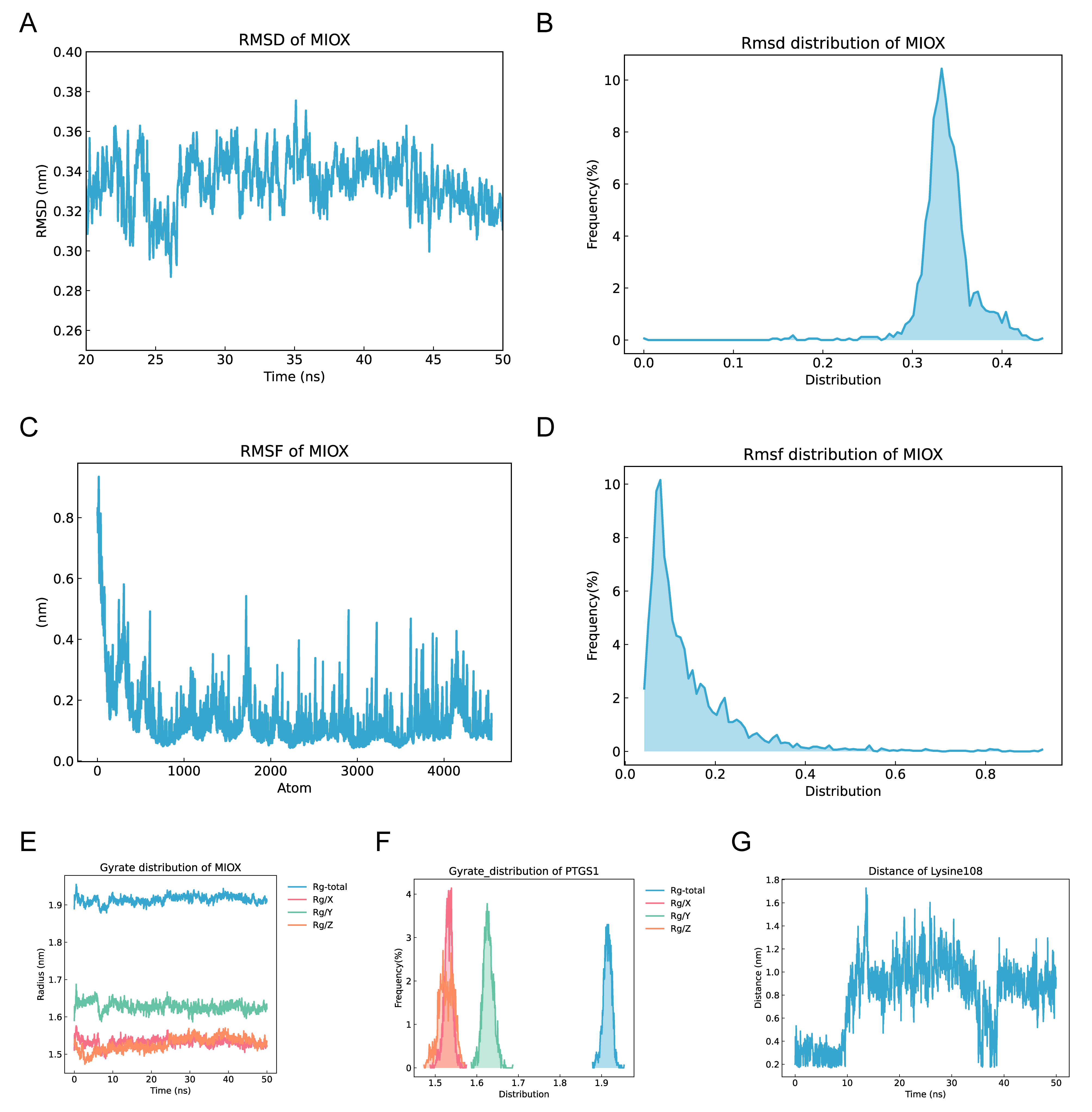

Supplement: Supplementary file 9 — Supplementary Material 9: Figure S2 MDs of MIOX and perfluorooctanoic acid (A-B) RMSD curve of MIOX protein. (C-D) RMSF plot of amino acid flexibility and exercise intensity of MIOX protein. (E–F) RoG plot of MIOX protein. (G) dynamic distance changes of key amino acids and ligands [file 41065_2025_385_MOESM9_ESM.tif]

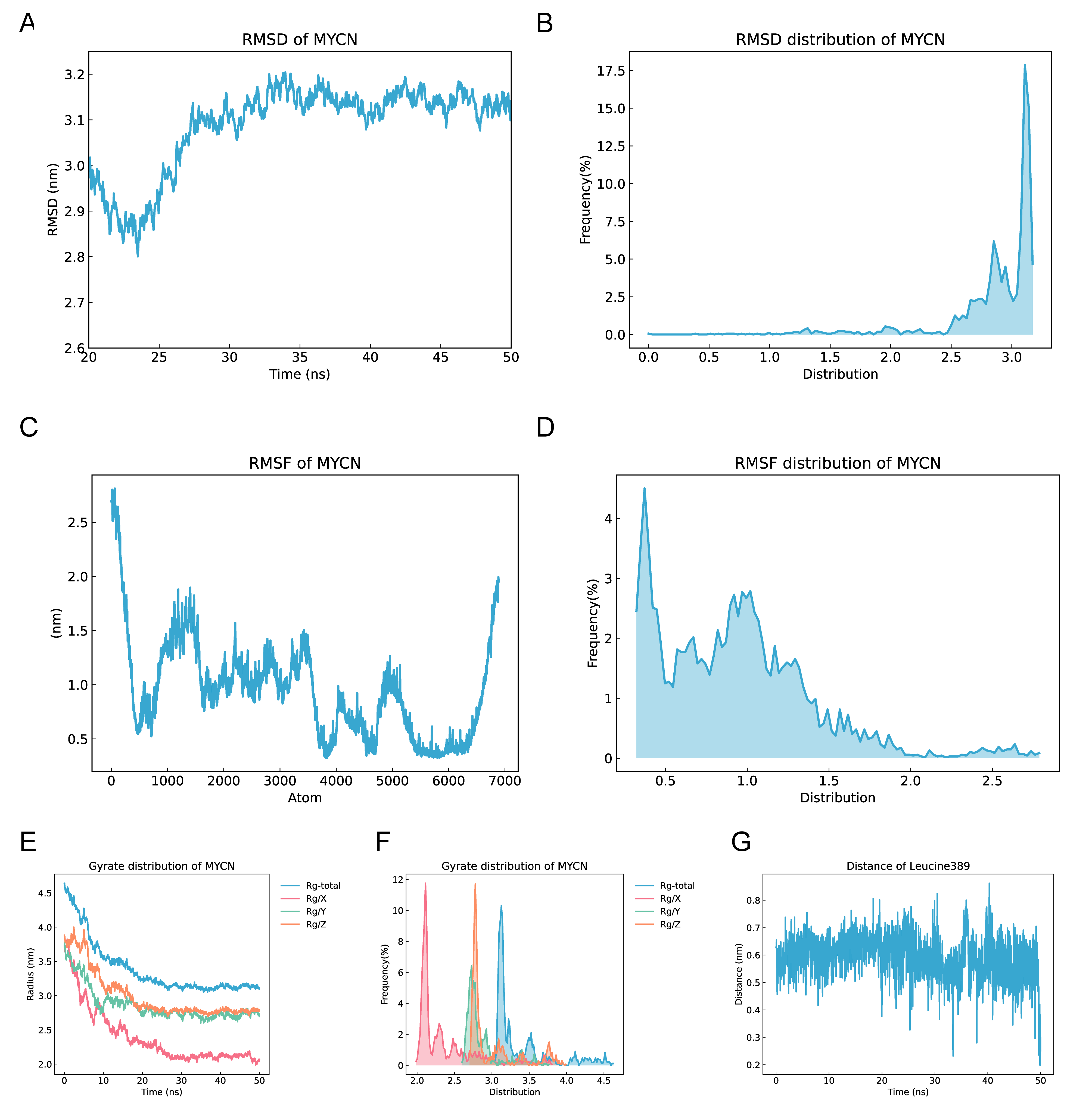

Supplement: Supplementary file 10 — Supplementary Material 10: Figure S3 MDs of MYCN and bisphenol A. (A-B) RMSD curve of MYCN protein. (C-D) RMSF plot of amino acid flexibility and exercise intensity of MYCN protein. (E–F) MYCN protein RoG plot. (G) dynamic distance changes of key amino acids and ligands [file 41065_2025_385_MOESM10_ESM.tif]

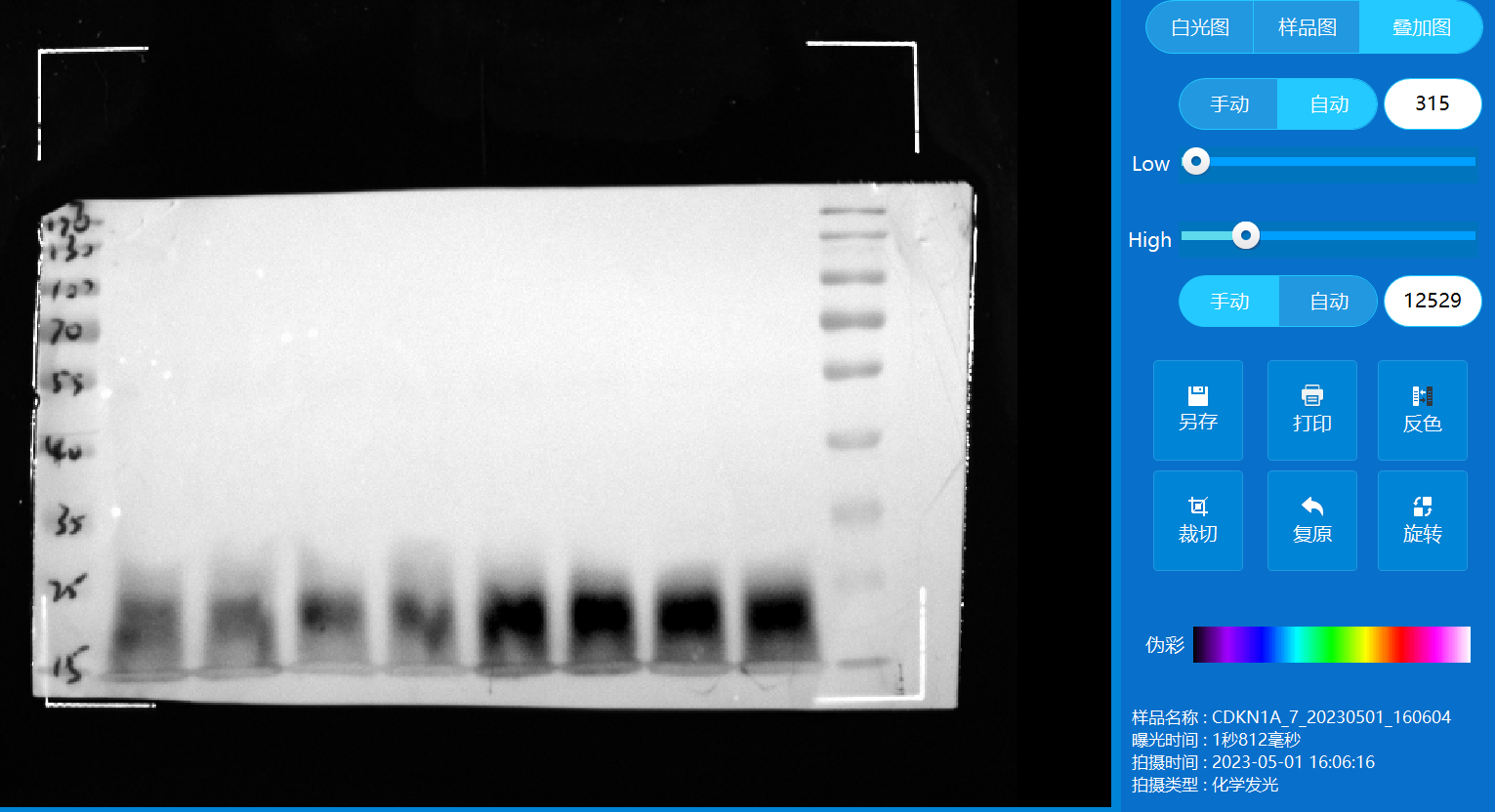

Supplement: Supplementary file 11 — Supplementary Material 11 [file 41065_2025_385_MOESM11_ESM.zip › supplementary file/Figure 10 uncropped Gels and Blots images/CDKN1A_7_20230501_160604_00.01.812_8bitú¿exposure time 1s812msú⌐.tif]

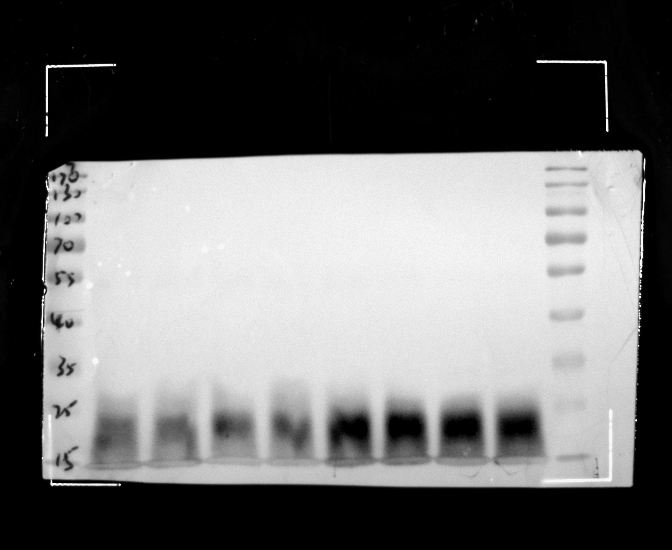

Supplement: Supplementary file 11 — Supplementary Material 11 [file 41065_2025_385_MOESM11_ESM.zip › supplementary file/Figure 10 uncropped Gels and Blots images/CDKN1A_7_20230501_160604_00.01.812_8bit.tif]

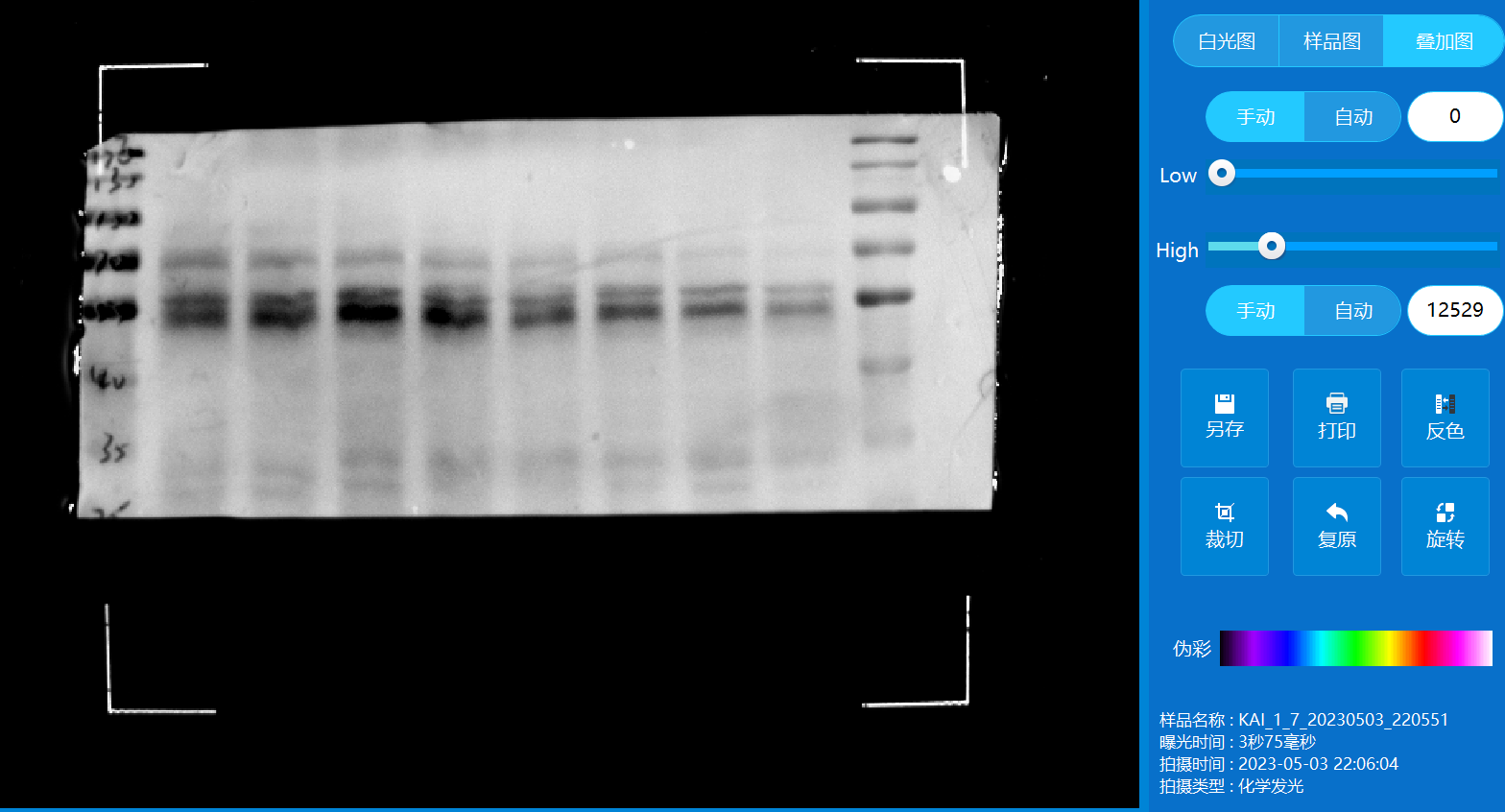

Supplement: Supplementary file 11 — Supplementary Material 11 [file 41065_2025_385_MOESM11_ESM.zip › supplementary file/Figure 10 uncropped Gels and Blots images/KAI_1_7_20230503_220551_00.03.075_8bit(0)(exposure time 3s75ms).tif]

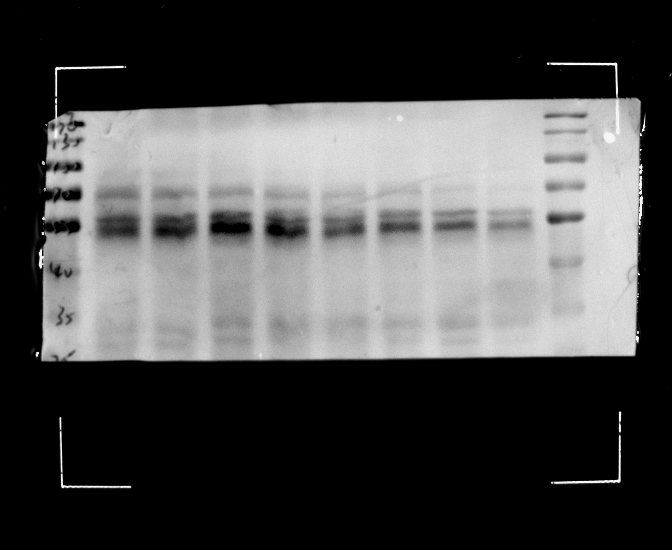

Supplement: Supplementary file 11 — Supplementary Material 11 [file 41065_2025_385_MOESM11_ESM.zip › supplementary file/Figure 10 uncropped Gels and Blots images/KAI_1_7_20230503_220551_00.03.075_8bit(0).tif]

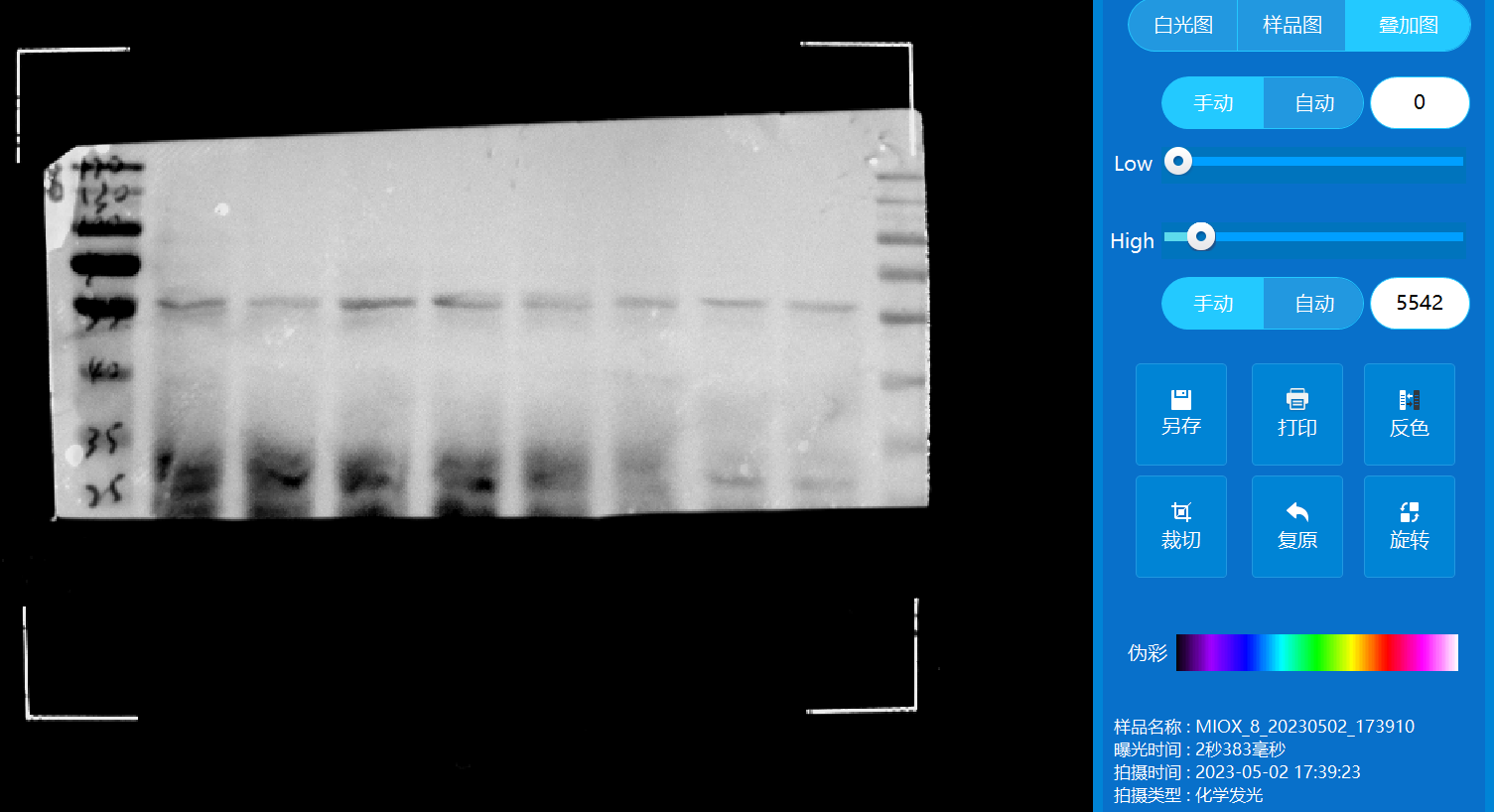

Supplement: Supplementary file 11 — Supplementary Material 11 [file 41065_2025_385_MOESM11_ESM.zip › supplementary file/Figure 10 uncropped Gels and Blots images/MIOX_8_20230502_173910_00.02.383_8bit(2)(exposure time 2s283ms).tif]

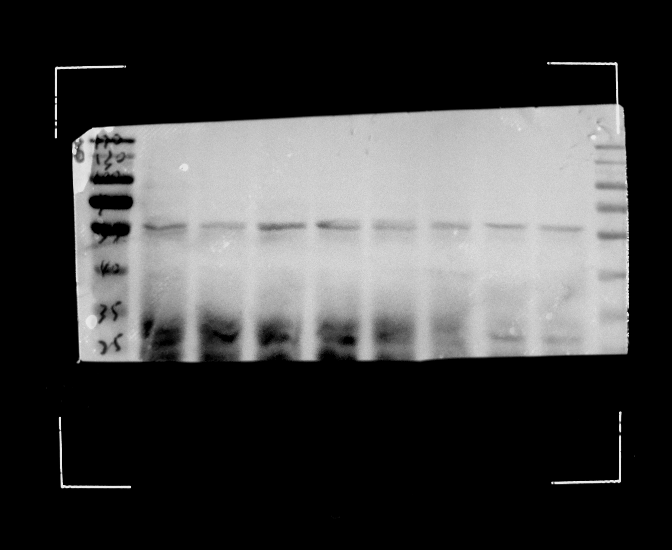

Supplement: Supplementary file 11 — Supplementary Material 11 [file 41065_2025_385_MOESM11_ESM.zip › supplementary file/Figure 10 uncropped Gels and Blots images/MIOX_8_20230502_173910_00.02.383_8bit(2).tif]

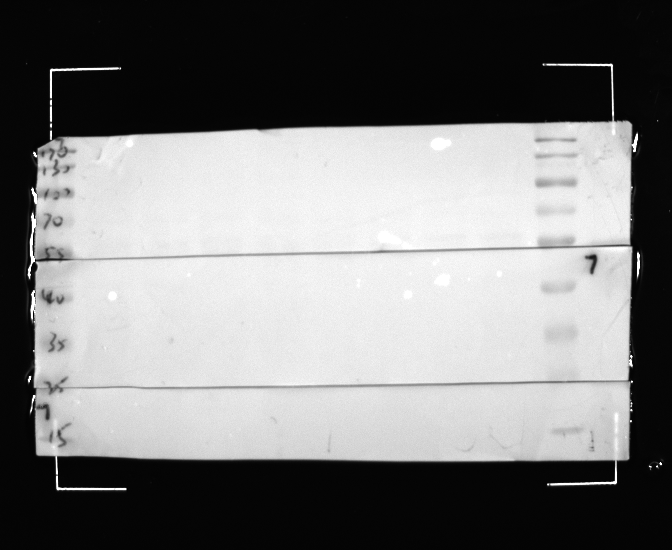

Supplement: Supplementary file 11 — Supplementary Material 11 [file 41065_2025_385_MOESM11_ESM.zip › supplementary file/Figure 10 uncropped Gels and Blots images/MYCNí¬í¬7_full gel_8bit.tif]

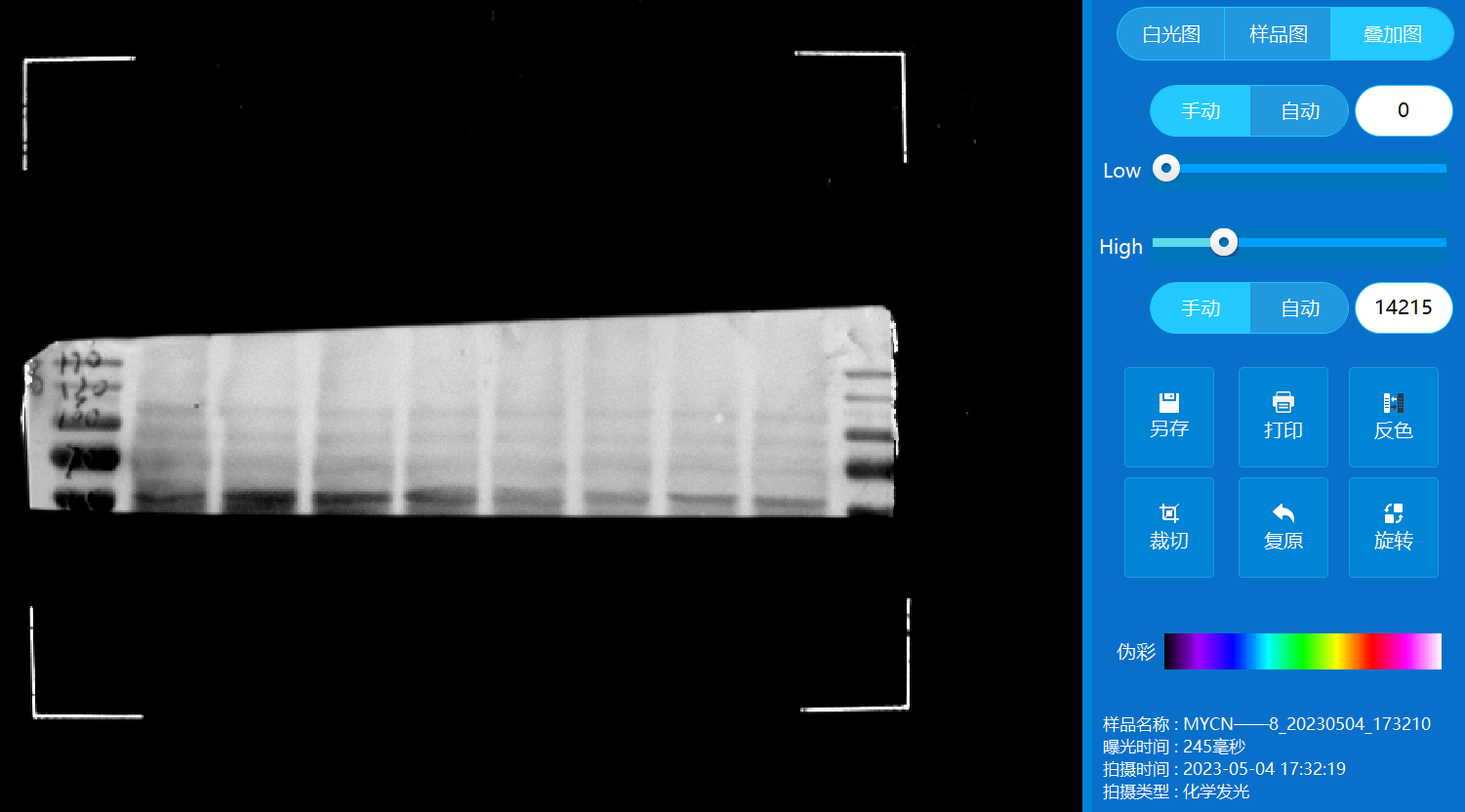

Supplement: Supplementary file 11 — Supplementary Material 11 [file 41065_2025_385_MOESM11_ESM.zip › supplementary file/Figure 10 uncropped Gels and Blots images/MYCNí¬í¬8_20230504_173210_00.00.245_8bit(exposure time 245ms).tif]

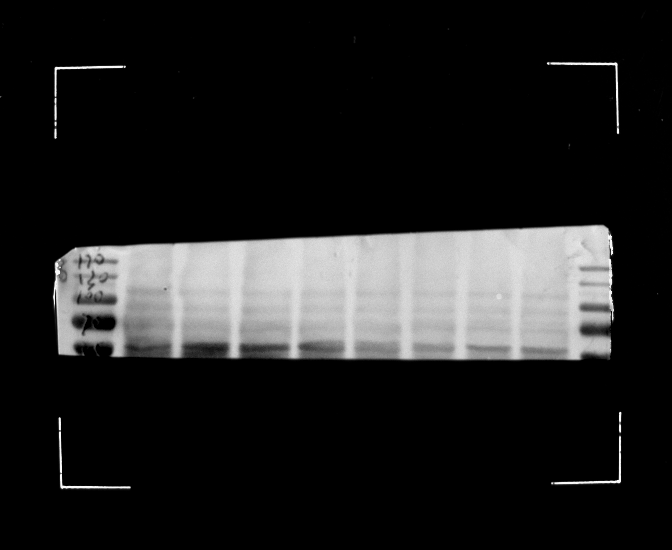

Supplement: Supplementary file 11 — Supplementary Material 11 [file 41065_2025_385_MOESM11_ESM.zip › supplementary file/Figure 10 uncropped Gels and Blots images/MYCNí¬í¬8_20230504_173210_00.00.245_8bit.tif]

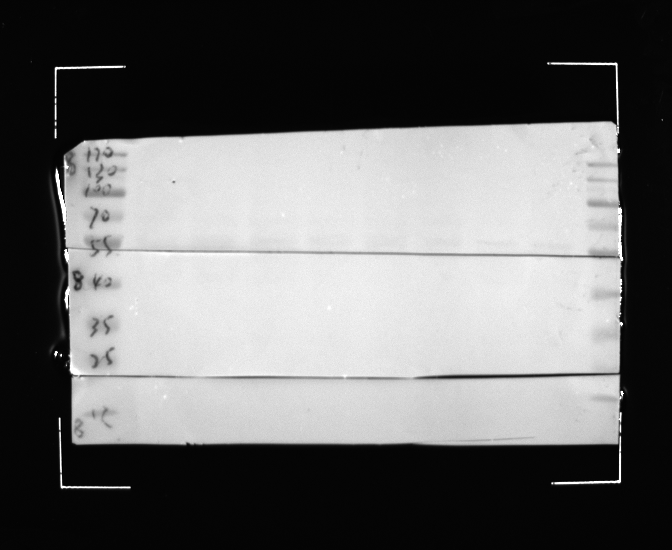

Supplement: Supplementary file 11 — Supplementary Material 11 [file 41065_2025_385_MOESM11_ESM.zip › supplementary file/Figure 10 uncropped Gels and Blots images/MYCNí¬í¬8_full gel_8bit.tif]

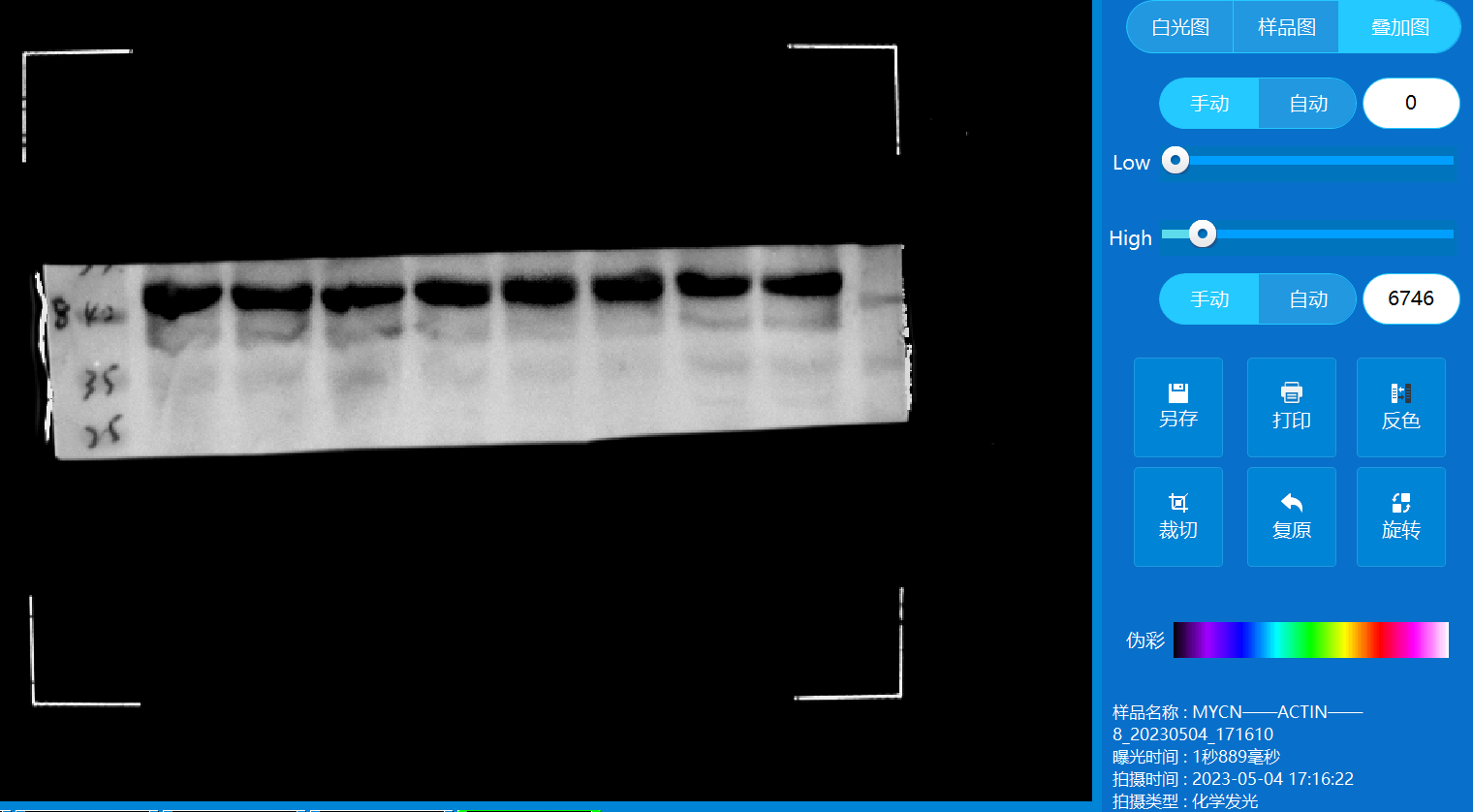

Supplement: Supplementary file 11 — Supplementary Material 11 [file 41065_2025_385_MOESM11_ESM.zip › supplementary file/Figure 10 uncropped Gels and Blots images/MYCNí¬í¬ACTINí¬í¬8_20230504_171610_00.01.889_8bit(3)(exposure time 1s889ms).tif]

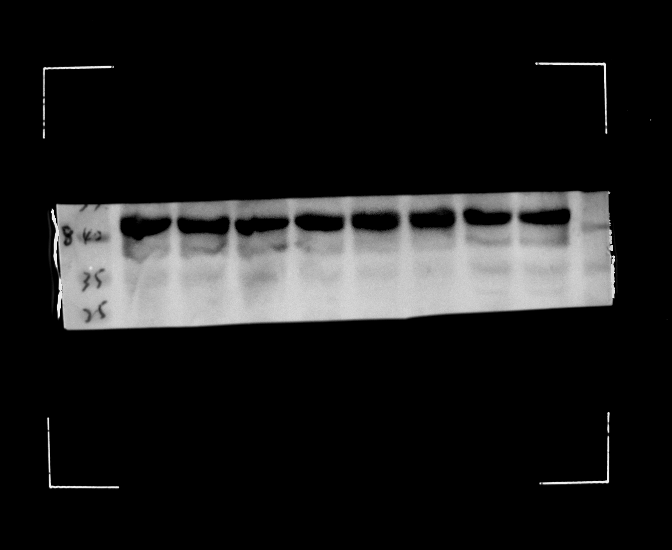

Supplement: Supplementary file 11 — Supplementary Material 11 [file 41065_2025_385_MOESM11_ESM.zip › supplementary file/Figure 10 uncropped Gels and Blots images/MYCNí¬í¬ACTINí¬í¬8_20230504_171610_00.01.889_8bit(3).tif]

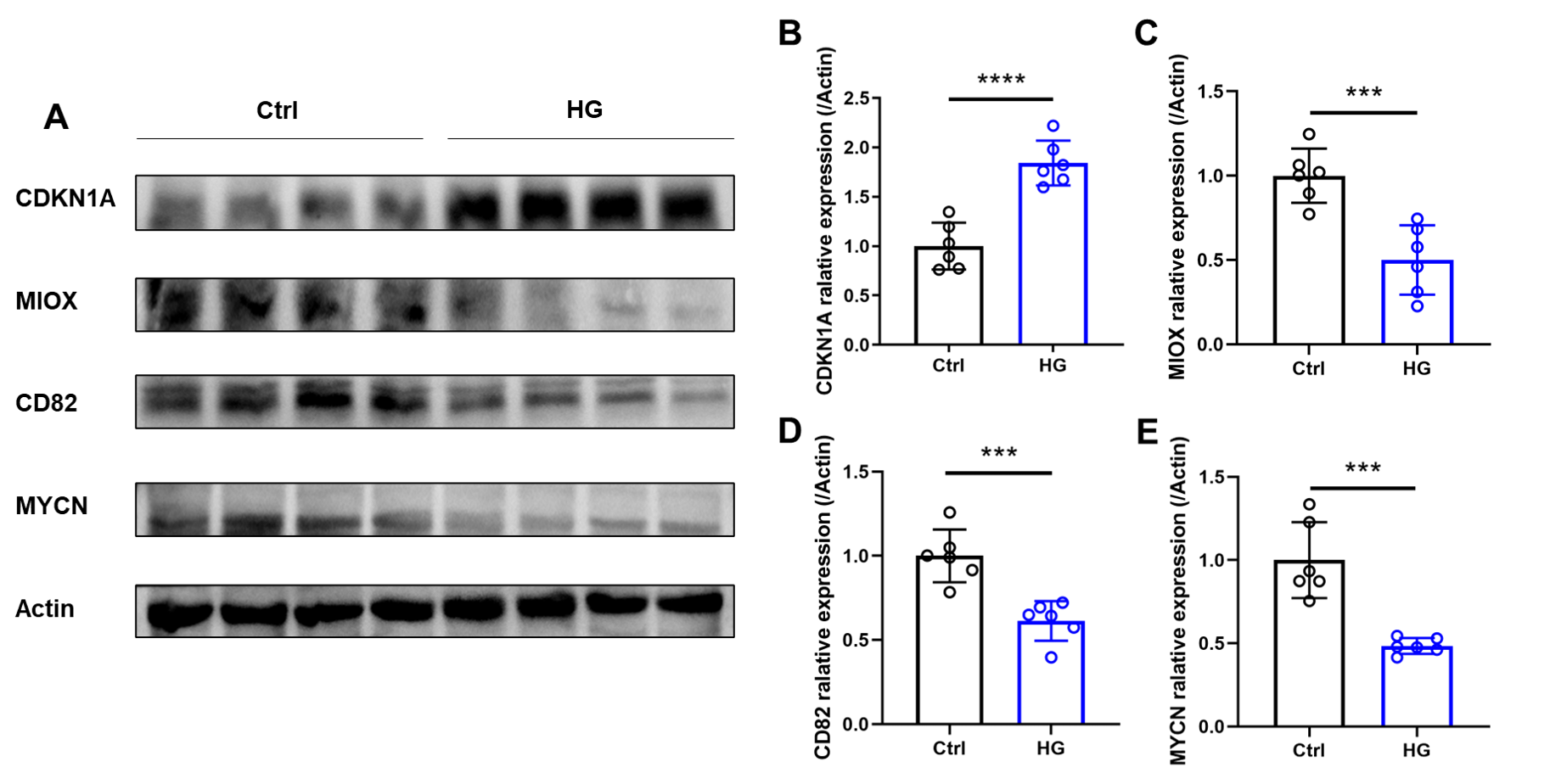

Supplement: Supplementary file 11 — Supplementary Material 11 [file 41065_2025_385_MOESM11_ESM.zip › supplementary file/Figure 10.tif]
