# Supplementary material for: Identification of biomarkers and mechanism exploration of ferroptosis related genes regulated by m6A in type 2 diabetes mellitus
Source: Hereditas. 2025 Feb 18;162:24. doi: 10.1186/s41065-025-00385-9 (PMC11834627; doi:10.1186/s41065-025-00385-9)
Supplement: Supplementary file 11 — Supplementary Material 11 [file 41065_2025_385_MOESM11_ESM.zip › supplementary file/Instruction of KAI 1 (G-10) sc-518002.pdf]

SANTA CRUZ BIOTECHNOLOGY, INC.

KAI 1 (G-10): sc-518002

BACKGROUND

The transmembrane 4 superfamily (TM4SF) is a family of leukocyte surface glycoproteins that presumably cross the cell membrane four times. These proteins may be involved in transmembrane signal transduction regulation of cell proliferation, differentiation and motility. Members of this family, which include CD9, CD37, CD53, CD63, CD82 and TAPA-1, share significant sequence homology and an extracellular N-glycosylated domain, implicating these proteins as metastasis suppressors. Only three members of this family have been correlated with metastasis: CD9, CD63 and CD82, also known as KAI 1. KAI 1 is evolutionarily conserved and expressed in a broad range of human tissues, but exhibits reduced expression in human cell lines derived from metastatic prostate tumors. It has been suggested that decreased KAI 1 expression may be involved in the malignant progression of prostate and perhaps other cancers.

CHROMOSOMAL LOCATION

Genetic locus: CD82 (human) mapping to 11p11.2.

SOURCE

KAI 1 (G-10) is a mouse monoclonal antibody raised against amino acids 95-267 of KAI 1 of human origin.

PRODUCT

Each vial contains 200 µg IgG_1 kappa light chain in 1.0 ml of PBS with < 0.1% sodium azide and 0.1% gelatin. Also available as TransCruz reagent for ChIP application, sc-518002 X, 200 µg/0.1 ml.

KAI 1 (G-10) is available conjugated to agarose (sc-518002 AC), 500 µg/0.25 ml agarose in 1 ml, for IP; to HRP (sc-518002 HRP), 200 µg/ml, for WB, IHC(P) and ELISA; to either phycoerythrin (sc-518002 PE), fluorescein (sc-518002 FITC), Alexa Fluor® 488 (sc-518002 AF488), Alexa Fluor® 546 (sc-518002 AF546), Alexa Fluor® 594 (sc-518002 AF594) or Alexa Fluor® 647 (sc-518002 AF647), 200 µg/ml, for WB (RGB), IF, IHC(P) and FCM; and to either Alexa Fluor® 680 (sc-518002 AF680) or Alexa Fluor® 790 (sc-518002 AF790), 200 µg/ml, for Near-Infrared (NIR) WB, IF and FCM.

APPLICATIONS

KAI 1 (G-10) is recommended for detection of KAI 1 of human origin by Western Blotting (starting dilution 1:100, dilution range 1:100-1:1000), immunoprecipitation [1-2 µg per 100-500 µg of total protein (1 ml of cell lysate)], immunofluorescence (starting dilution 1:50, dilution range 1:50-1:500), immunohistochemistry (including paraffin-embedded sections) (starting dilution 1:50, dilution range 1:50-1:500) and solid phase ELISA (starting dilution 1:30, dilution range 1:30-1:3000).

Suitable for use as control antibody for KAI 1 siRNA (h): sc-35734, KAI 1 shRNA Plasmid (h): sc-35734-SH and KAI 1 shRNA (h) Lentiviral Particles: sc-35734-V.

KAI 1 (G-10) X TransCruz antibody is recommended for ChIP assays.

Molecular Weight of KAI 1: 46 kDa.

Positive Controls: Jurkat whole cell lysate: sc-2204, K-562 whole cell lysate: sc-2203 or HL-60 whole cell lysate: sc-2209.

RECOMMENDED SUPPORT REAGENTS

To ensure optimal results, the following support reagents are recommended: 1) Western Blotting: use m-IgGκ BP-HRP: sc-516102 or m-IgGκ BP-HRP (Cruz Marker): sc-516102-CM (dilution range: 1:1000-1:10000), Cruz Marker™ Molecular Weight Standards: sc-2035, UltraCruz® Blocking Reagent: sc-516214 and Western Blotting Luminol Reagent: sc-2048. 2) Immunoprecipitation: use Protein A/G PLUS-Agarose: sc-2003 (0.5 ml agarose/2.0 ml). 3) Immunofluorescence: use m-IgGκ BP-FITC: sc-516140 or m-IgGκ BP-PE: sc-516141 (dilution range: 1:50-1:200) with UltraCruz® Mounting Medium: sc-24941 or UltraCruz® Hard-set Mounting Medium: sc-359850. 4) Immunohistochemistry: use m-IgGκ BP-HRP: sc-516102 with DAB, 50X: sc-24982 and Immunohistomount: sc-45086, or Organo/Limonene Mount: sc-45087.

DATA

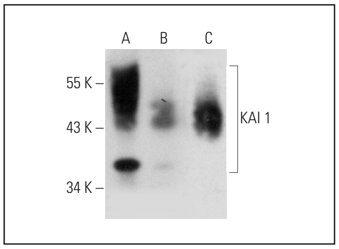

KAI 1 (G-10): sc-518002. Western blot analysis of KAI 1 expression in Jurkat (**A**), K-562 (**B**) and HL-60 (**C**) whole cell lysates.

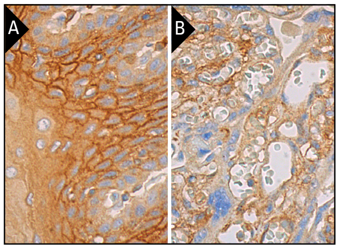

KAI 1 (G-10): sc-518002. Immunoperoxidase staining of formalin fixed, paraffin-embedded human esophagus tissue showing membrane and cytoplasmic staining of squamous epithelial cells (**A**). Immunoperoxidase staining of formalin fixed, paraffin-embedded human placenta tissue showing membrane and cytoplasmic staining of endothelial cells (**B**).

SELECT PRODUCT CITATIONS

1. Feng, Y., et al. 2020. Characterizing pancreatic β-cell heterogeneity in the streptozotocin model by single-cell transcriptomic analysis. Mol. Metab. 37: 100982.

STORAGE

Store at 4° C, **DO NOT FREEZE**. Stable for one year from the date of shipment. Non-hazardous. No MSDS required.

RESEARCH USE

For research use only, not for use in diagnostic procedures.

PROTOCOLS

See our web site at www.scbt.com for detailed protocols and support products.

Alexa Fluor® is a trademark of Molecular Probes, Inc., Oregon, USA
